# Treating the placenta to prevent adverse effects of gestational hypoxia on fetal brain development

**DOI:** 10.1038/s41598-017-06300-1

**Published:** 2017-08-22

**Authors:** Tom J. Phillips, Hannah Scott, David A. Menassa, Ashleigh L. Bignell, Aman Sood, Jude S. Morton, Takami Akagi, Koki Azuma, Mark F. Rogers, Catherine E. Gilmore, Gareth J. Inman, Simon Grant, Yealin Chung, Mais M. Aljunaidy, Christy-Lynn Cooke, Bruno R. Steinkraus, Andrew Pocklington, Angela Logan, Gavin P. Collett, Helena Kemp, Peter A. Holmans, Michael P. Murphy, Tudor A. Fulga, Andrew M. Coney, Mitsuru Akashi, Sandra T. Davidge, C. Patrick Case

**Affiliations:** 1 School of Clinical Sciences, University of Bristol, Southmead Hospital, Bristol, BS10 5NB UK; 2 Department of Obstetrics and Gynecology and Women and Children’s Health Research Institute, University of Alberta, Edmonton, Alberta Canada; 3 Department of Applied Chemistry, Graduate School of Engineering, Osaka University, Osaka, 565-0871 Japan; 4 Graduate School of Frontier Biosciences, Osaka University, Osaka, 565-0871 Japan; 5 Intelligent Systems Laboratory, University of Bristol, Merchant Venturers Building, Bristol, BS8 1UB UK; 6 Division of Cancer Research, Jacqui Wood Cancer Centre, University of Dundee, Dundee, DD1 9SY UK; 7 Department of Obstetrics, Southmead Hospital, Bristol, BS10 5NB UK; 8 School of Social and Community Medicine, University of Bristol, Southmead Hospital, Bristol, BS10 5NB UK; 9 Weatherall Institute of Molecular Medicine, Radcliffe Department of Medicine, University of Oxford, Oxford, OX3 9DS UK; 10 Institute of Psychological Medicine and Clinical Neurosciences and MRC Centre for Neuropsychiatric Genetics and Genomics, Cardiff University School of Medicine, Cardiff, CF24 4HQ UK; 11 MRC Mitochondrial Biology Unit, Wellcome Trust/MRC Building, Cambridge, CB2 0XY UK; 12 Nuffield Department of Obstetrics & Gynaecology, University of Oxford, John Radcliffe Hospital, Oxford, OX3 9DU UK; 13 Department of Clinical Biochemistry, Pathology Sciences Laboratory, Southmead Hospital, Bristol, BS10 5NB UK; 14 Institute of Clinical Sciences, College of Medical and Dental Sciences, University of Birmingham, Birmingham, B15 2TT UK; 15 Department of Physiology, University of Alberta, Edmonton, Alberta Canada

## Abstract

Some neuropsychiatric disease, including schizophrenia, may originate during prenatal development, following periods of gestational hypoxia and placental oxidative stress. Here we investigated if gestational hypoxia promotes damaging secretions from the placenta that affect fetal development and whether a mitochondria-targeted antioxidant MitoQ might prevent this. Gestational hypoxia caused low birth-weight and changes in young adult offspring brain, mimicking those in human neuropsychiatric disease. Exposure of cultured neurons to fetal plasma or to secretions from the placenta or from model trophoblast barriers that had been exposed to altered oxygenation caused similar morphological changes. The secretions and plasma contained altered microRNAs whose targets were linked with changes in gene expression in the fetal brain and with human schizophrenia loci. Molecular and morphological changes *in vivo* and *in vitro* were prevented by a single dose of MitoQ bound to nanoparticles, which were shown to localise and prevent oxidative stress in the placenta but not in the fetus. We suggest the possibility of developing preventative treatments that target the placenta and not the fetus to reduce risk of psychiatric disease in later life.

## Introduction

The hypothesis for the fetal origin of adult diseases was first proposed in 1990 by David Barker^1^. Since then, evidence has accumulated lending credence to the hypothesis that some neuropsychiatric diseases that become symptomatic during adolescence or in adulthood have a neurodevelopmental origin^2, 3^. Furthermore, psychological disorders such as schizophrenia, attention deficit hyperactivity disorder and autism have been associated with episodes of altered oxygen during pregnancy, as early as the end of the first trimester^3–7^. For schizophrenia in particular, a two-hit hypothesis has been proposed where the first ‘hit’ occurs *in utero*, disrupting neuroanatomical wiring and thereby increasing susceptibility to a second hit in later life, which triggers the disease symptoms^8^.

The neuropathology of neuropsychiatric disorders has been studied extensively and pathological findings generally overlap between the various disorders. For example, dendrite dysgenesis^9, 10^ and dendritic spine pathology^11–16^ of cortical neurons has been often reported in multiple brain regions including temporal, frontal and occipital cortices from autism, schizophrenia and mental retardation patients. Changes in the catecholamine system including altered tyrosine hydroxylase activity^17, 18^, loss of parvalbumin-positive neurons^19, 20^, and dysfunction of NMDA receptors^9, 17, 21, 22^ have been observed in post-mortem brains of patients with schizophrenia and autism spectrum disorders as well as in animal models. Finally, abnormal astrocyte number or function has also been proposed as a critical change in autism and schizophrenia brains^23–26^.

The mechanisms by which adverse events during gestation can alter fetal development are not well understood. The placenta, which presents a double-layered interface between maternal and fetal blood during the first trimester, may play an important role^21, 27–32^. During very early development the fetal brain’s supply of the transmitter serotonin^33^, and possibly others^34^, is derived from the placenta. Obstetric challenge in the form of maternal inflammation, can alter this signalling from the placenta to the brain and disrupt axonal outgrowth of 5HT-positive neurons within the fetal forebrain^35^. Furthermore preeclampsia, a pregnancy complication associated with placental insufficiency and abnormal oxygenation of the placenta, is a risk factor for the development of neuropsychiatric disease^36–39^.

We have shown previously that induction of oxidative stress in the human placenta or a simple model of it, a trophoblast cell barrier, causes it to release molecules that elicit DNA damage in human fibroblasts and human embryonic stem cells^40–42^. Secretions from barriers exposed to hypoxia also caused morphological, electrophysiological and receptor-related changes to embryonic neurones and astrocytes, both in culture and in the developing brain *in vivo* ^43^. We noted that soluble drugs such as MitoQ, a mitochondrial antioxidant, Gap26, which blocks connexin hemichannels and gap junctions, PPADS, a purinergic receptor antagonist, and C17, an antagonist of pannexin channels, could inhibit intercellular signalling between the layers of the barrier and prevent the secretion of DNA damaging factors from the barrier^40, 41^. These observations suggest that secreted placental factors may be candidate effectors for fetal programming of disease. They present an opportunity to treat the placenta via the mother to prevent harmful signalling to the fetus.

Few drugs are used in pregnancy because of their potential adverse effects on the fetus, should they pass through the placenta. Previously we showed that nanoparticles accumulate in the top layer of a bilayered trophoblast barrier and do not pass through it^41, 42^. Coupling a drug that stops oxidative stress in the placenta to nanoparticles could therefore prevent passage of the drug through the placenta and to the fetus. Here we investigate the potential of treating the placenta with a nanoparticle-bound MitoQ prior to a hypoxic insult to prevent damaging signalling that could affect neurodevelopment in a rat model of gestational hypoxia *in vivo*.

## Results

**Figure 1:**
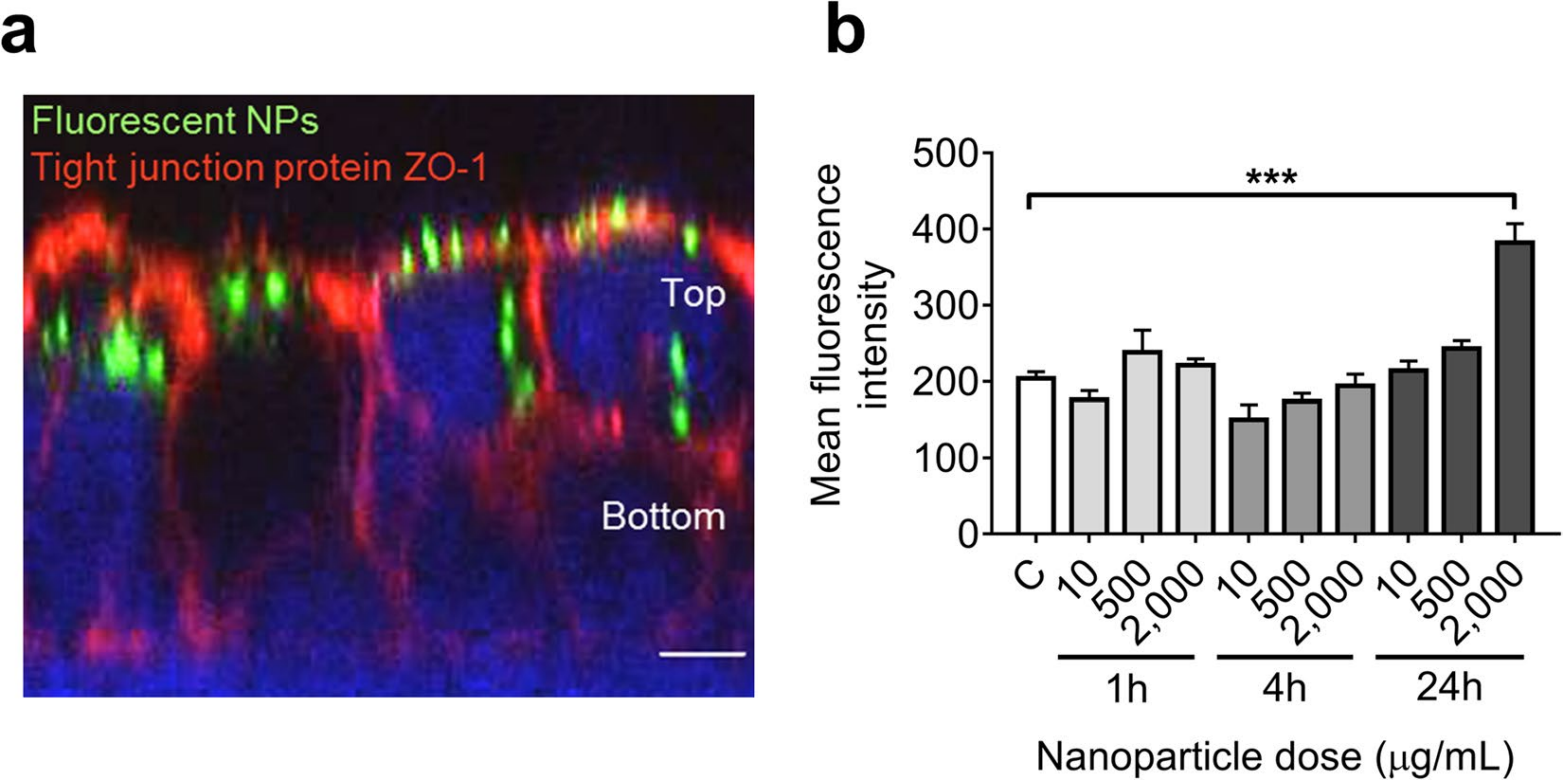
Characterisation of MitoQ-NPs. (**a**) Confocal images of bilayered BeWo barriers 24 h after application of 2 mg/mL fluorescent NPs (green) to the top of the barrier. Tight junction protein ZO-1 is labelled in red, nuclei in blue. Scale bar = 10 μm. (**b**) Levels of fluorescence detected in the tissue culture medium below the bilayered BeWo barriers up to 24 h after application of different doses of NPs to the top of the barrier (*n* = 2). ***p < 0.001 as determined by *post*-*hoc* testing following one-way ANOVA with Bonferroni correction for multiple comparisons.

Antioxidant MitoQ was successfully coupled to nanoparticles (NPs) (Supplementary Data; Supplementary Fig. S1) and tested *in vitro* on bilayered barriers of BeWo cells^41^, a choriocarcinoma cell line. MitoQ-NPs were most effective at reducing the effects of *in vitro* hypoxia, compared to NP-bound Gap26 or PPADS (Supplementary Fig. S2). When NPs were applied to BeWo barriers for up to 24 h, they were predominantly located in the top layer of the barrier (Fig. 1a)^40^. There was no evidence of passage of NPs, or release of MitoQ, across the barriers into the media below except at a very high NP dose (2 mg/ml) (Fig. 1c; Supplementary Table S1). For subsequent *in vivo* experiments a 2580-fold lower dose was selected, at which NPs were not observed to cross the *in vitro* barrier.

We investigated if treatment with MitoQ-NPs would be able to prevent placental oxidative stress, altered signalling from the placenta and neuroanatomical changes in the offspring in an established *in vivo* model of gestational hypoxia^44^. Briefly, pregnant rats were exposed to 11% oxygen for the last 6 days of pregnancy. This exposure reduces birth-weight and is known to cause abnormal fetal cardiovascular programming^30, 45^. We used a single dose of MitoQ-NPs, injected intravenously into the dam at the start of the hypoxic exposure, as the least invasive procedure.

### Effects of MitoQ-NPs on placenta and fetus

**Figure 2:**
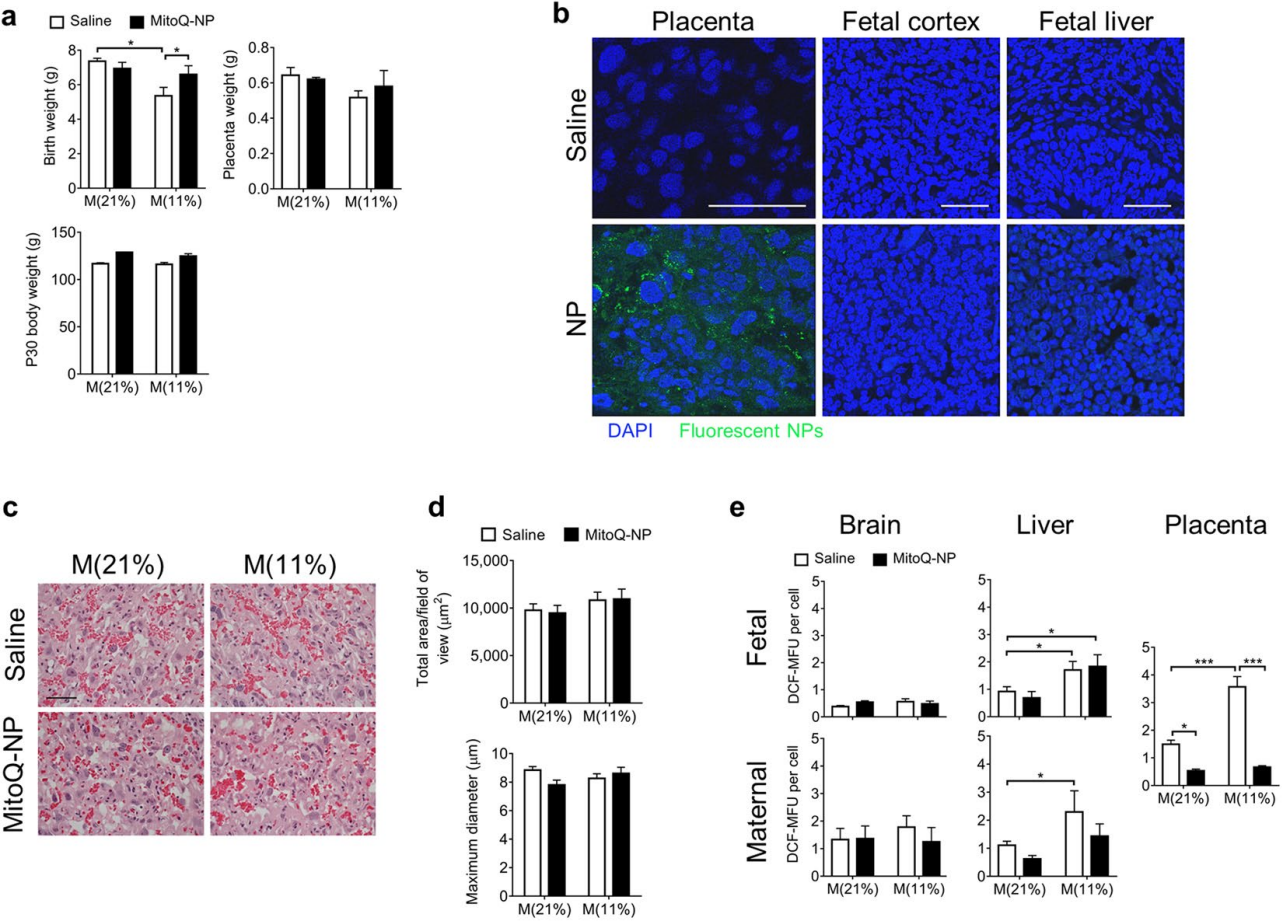
Effects of MitoQ-NPs on the rat *in vivo*. (**a**) Birth-weight (top left; *n* = 6 litters), placenta weight (top right; *n* = 6 litters) and body weight at P30 (bottom; *n* = 24 individuals) of offspring exposed to maternal normoxia, M(21%), or hypoxia, M(11%), preceded by maternal administration of saline or MitoQ-NPs. (**b**) Confocal images of rat placental labyrinth, fetal cortex and fetal liver at GD16 after maternal injection with saline or fluorescent NPs (green), counterstained with DAPI (blue), scale bar = 100 μm. (**c**) Light micrographs showing the gross histology (H&E stain) of the placental labyrinth at GD20 following *in vivo* normoxia or hypoxia with maternal saline or MitoQ-NP injection (scale bar = 2 μm). (**d**) Analysis of total area per field of view (top) and maximum diameter (bottom) of placental blood vessels after gestational hypoxia combined with maternal injection of saline or MitoQ-NPs (*n* = 3 placenta per condition, each from a different dam). (**e**) Levels of fluorescent dichlorofluorescein (DCF) as a measure of reactive oxygen species and thus oxidative stress in fetal brain (left, top) and liver (middle, top), maternal brain (left, bottom) and liver (middle, bottom) and placenta (right) after exposure to altered oxygen *in vivo*, with or without maternal MitoQ-NP injection (biological replicates: fetal and placental samples, *n* = 6; maternal samples, *n* = 3). Additional significant differences for ‘placenta’ graph: M(21%) + saline vs M(11%) + MitoQ-NP, *p* < 0.05; M(21%) + MitoQ-NP vs M(11%) + saline, *p* < 0.001. *p < 0.05, ***p < 0.001 as determined by *post*-*hoc* testing following ANOVA.

As expected^30, 45^, birth-weights were decreased following maternal hypoxia (Fig. 2a). Maternal MitoQ-NP injection rescued over 60% of this deficit. Hypoxia and MitoQ-NPs had no effect on body weight at P30, placental weight or brain weight (Fig. 2a; Supplementary Fig. S3).

We investigated if NPs could reach the placenta *in vivo* and whether maternal hypoxia or MitoQ-NP injection caused changes to the placenta. NPs were detected within the placenta, most prevalently in the labyrinth but also in the junctional zone (Fig. 2b). The NPs were particularly found in cytotrophoblasts, which face the maternal circulation, and less commonly seen in syncytiotrophoblast cells, which face the fetus. NPs were not found in the fetal brain or in thoracic or abdominal tissues including liver (Fig. 2b). NPs were detected in the maternal liver, particularly in Kupffer cells and hepatocytes (Supplementary Fig. S3). Areas of NP localisation were observed sparsely throughout the maternal brain. Within these areas, fluorescence was mainly detected within neuronal processes (Supplementary Fig. S3).

Neither maternal hypoxia nor NP injection caused a change in placental fine structure (Fig. 2c) or in the width of labyrinth or decidua (Supplementary Fig. S3). Nor was the fetal capillary network altered in the labyrinth (Fig. 2d; Supplementary Fig. S3). Levels of oxidative stress were significantly increased in the labyrinth and junctional zones of the placenta following gestational hypoxia. Treatment with MitoQ-NPs significantly reduced levels of oxidative stress (Fig. 2e; Supplementary Fig. S3). Analysis of maternal liver and fetal liver showed elevated levels of oxidative stress following maternal hypoxia. After MitoQ treatment increased oxidative stress was still observed in fetal liver but not in maternal liver (Fig. 2e). No changes in oxidative stress were detected in the fetal brain after maternal hypoxia or NP treatment (Fig. 2e; Supplementary Fig. S3).

These observations suggest that the NPs injected into the maternal circulation reduce oxidative stress in maternal tissues and placenta but not in fetal tissues following gestational hypoxia. Moreover, they rescue fetal birth-weight although they do not reach the fetus.

### Effects of MitoQ-NPs on placental secretions

Several authors have stressed the importance of the placenta, as the interface between mother and fetus, in fetal programming^21, 27–32^. We hypothesised that secretions from the placental barrier in response to hypoxia, as observed previously *in vitro* ^43^, might play a role in fetal development *in vivo*. By targeting oxidative stress in the placenta, MitoQ-NPs may prevent potentially damaging signalling from the placenta. We tested the hypothesis that MitoQ-NPs could alter the secretion of molecules from the placenta by investigating microRNAs (miRNAs), proteins including bone morphogenetic proteins (BMPs) and amino acids, all of which have potential relevance to fetal development^46–49^, psychiatric diseases^50–52^ and placental hypoxia^53, 54^. Levels of these molecules were measured in fetal plasma and in culture medium conditioned *ex vivo* by placental tissue (‘rat placenta conditioned medium’), collected from rats exposed to gestational hypoxia and MitoQ-NP treatment. Additionally, we analysed culture medium conditioned *in vitro* by bilayered barriers of BeWo cells (‘BeWo barrier conditioned medium’) following exposure to altered oxygen and MitoQ-NPs.

**Figure 3:**
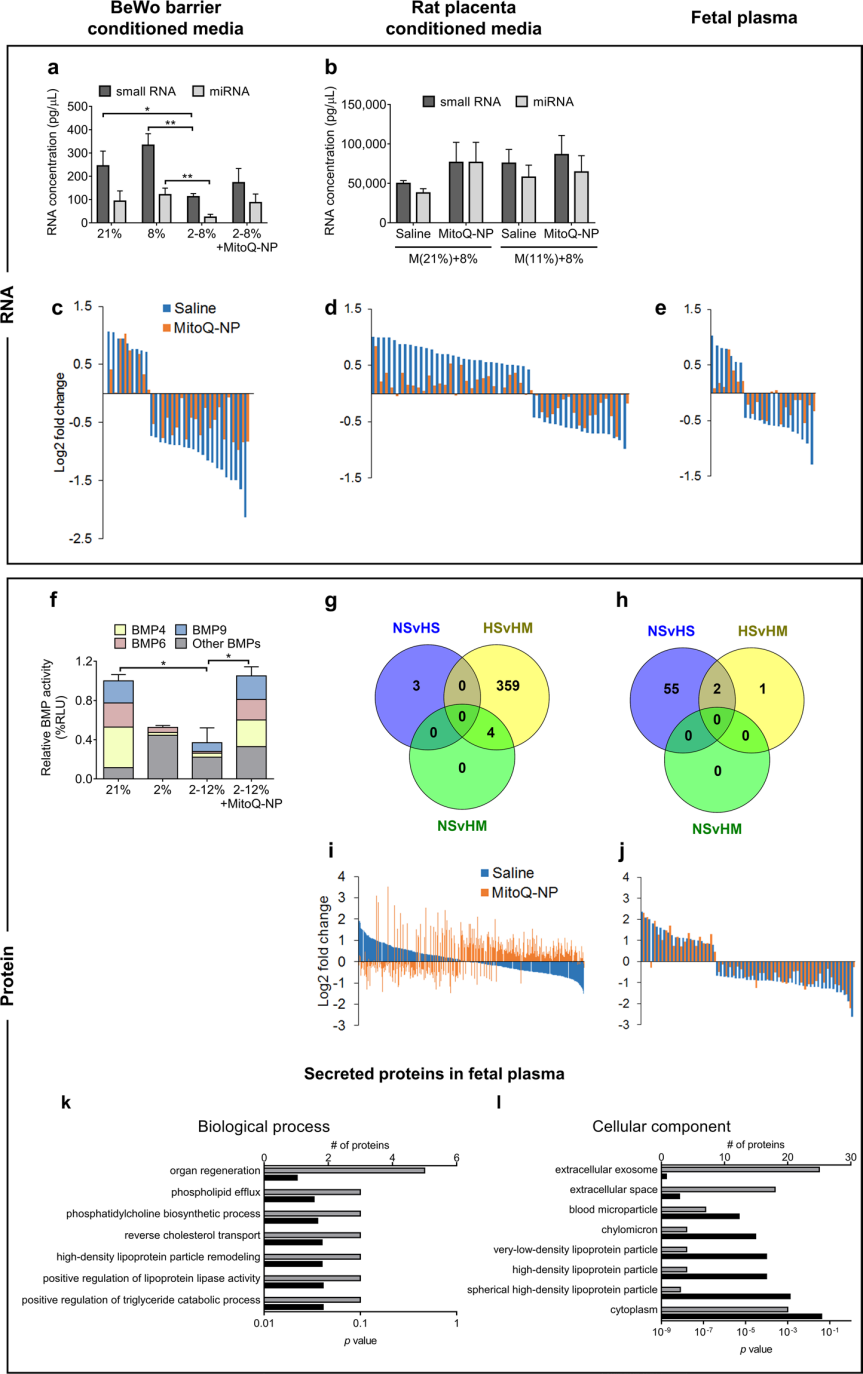
Effects of MitoQ-NPs on secreted molecules from BeWo barriers and placenta and into fetal blood. Levels of molecules in media conditioned by bilayered BeWo barriers (**a**,**c**,**f**) or rat placenta (**b**,**d**,**g**,**i**), and in fetal plasma (**e**,**h**,**j–l**). BeWo barriers had been exposed to 21% oxygen, to 8% oxygen or to 2% oxygen followed by 8% oxygen to mimic hypoxia-reoxygenation (2–8%), with or without application of MitoQ-NPs. Placentas and fetal plasma were collected from rats exposed to gestational normoxia, M(21%), or hypoxia, M(11%), after maternal administration of saline or MitoQ-NPs. Placentas were incubated *ex vivo* for 24 h at 8% oxygen before collection of conditioned medium. (**a**,**b**) Total levels of small RNAs and miRNAs. (**c–e**) Log2-fold changes of miRNAs with significantly (*p* < 0.05) different secretion under altered oxygen alone (blue) or altered oxygen plus MitoQ-NP application (orange), relative to control levels (normoxia + saline). (**f**) Relative activity of BMPs in relative luminescence unit (RLU), normalised to 21%. Significant differences not shown in graph: BMP4, 21%vs2%, *p* = 0.01, 21%vs2-12%, *p* = 0.04; Other BMPs, 21%vs2%, *p* = 0.004; 21%vs2-12%, *p* = 0.04. (**g**,**h**) Levels of significant (*p* < 0.05) differentially abundant proteins in rat placenta conditioned medium (**g**) and fetal plasma (**h**). Differences were analysed between: normoxia + saline and hypoxia + saline (NSvHS); hypoxia + saline and hypoxia + MitoQ-NPs (HSvHM); normoxia + saline and hypoxia + MitoQ-NPs (NSvHM). (**i**) Log2-fold changes of proteins detected in rat placenta-conditioned medium under altered oxygen alone (blue) or altered oxygen plus MitoQ-NP application (orange), relative to control (normoxia + saline). Proteins depicted showed significant differential abundance in the hypoxia + MitoQ-NP conditioned compared to hypoxia + saline. *n* = 3 biological replicates, each from a different litter. (**j**) Log2-fold changes of proteins with significantly different abundance in fetal plasma under altered oxygen alone (blue) or altered oxygen plus MitoQ-NP application (orange), relative to control levels. *n* = 4 for groups normoxia + saline, normoxia + MitoQ-NPs and hypoxia + MitoQ-NPs; all others, *n* = 3. (**k**,**l**) Gene ontology analysis of significant differentially abundant proteins in fetal plasma after exposure to maternal hypoxia compared to normoxia. Enrichment of biological processes (**k**) and cellular compartments (**l**) is shown. Grey bars represent number of proteins assigned to each gene ontology term and black bars show respective *p* value. *p < 0.05, **p < 0.01, Tukey’s *post*-*hoc* test following one-way or two-way ANOVA.

Changes to oxygen levels altered total levels of miRNAs, small RNAs and potentially small extracellular vesicles, such as exosomes, released from BeWo barriers (Fig. 3a; Supplementary Fig. S4). There was no change in total miRNA or small RNA in medium conditioned by rat placenta after gestational hypoxia (Fig. 3b; Supplementary Fig. S4). However in media conditioned by BeWo barriers or rat placenta and in fetal plasma there was a complex pattern of increased and decreased levels of individual miRNA (Fig. 3c–e; Supplementary Fig. S4). The application of MitoQ-NPs partially prevented these changes for the majority of differentially secreted miRNAs.

The pattern of secretion of BMPs from BeWo barriers was also altered following exposure to reduced oxygen (Fig. 3f). This was prevented by the MitoQ-NPs. The proteome profile of fetal plasma was changed in response to gestational hypoxia (Fig. 3h,j). Proteins with altered abundance were significantly enriched for exosomes and lipoproteins (Fig. 3k,l). No change in protein abundance was seen in the fetal plasma after maternal MitoQ-NP treatment (Fig. 3h,j). In contrast, in rat placenta conditioned media, there was little change in protein levels after gestational hypoxia but significant change after MitoQ-NP treatment (Fig. 3g,i).

Levels of amino acids in rat placenta conditioned media were not significantly altered in response to maternal hypoxia (Supplementary Tables S2–4; Supplementary Fig. S4).

Taken together these results indicate that gestational hypoxia selectively alters the secretion of miRNAs and proteins *in vivo*, *ex vivo* and *in vitro*. Maternal administration of MitoQ-NPs ‘normalises’ the miRNA secretion patterns in a global manner.

### Effects of MitoQ-NPs on gene expression in the offspring brain

**Figure 4:**
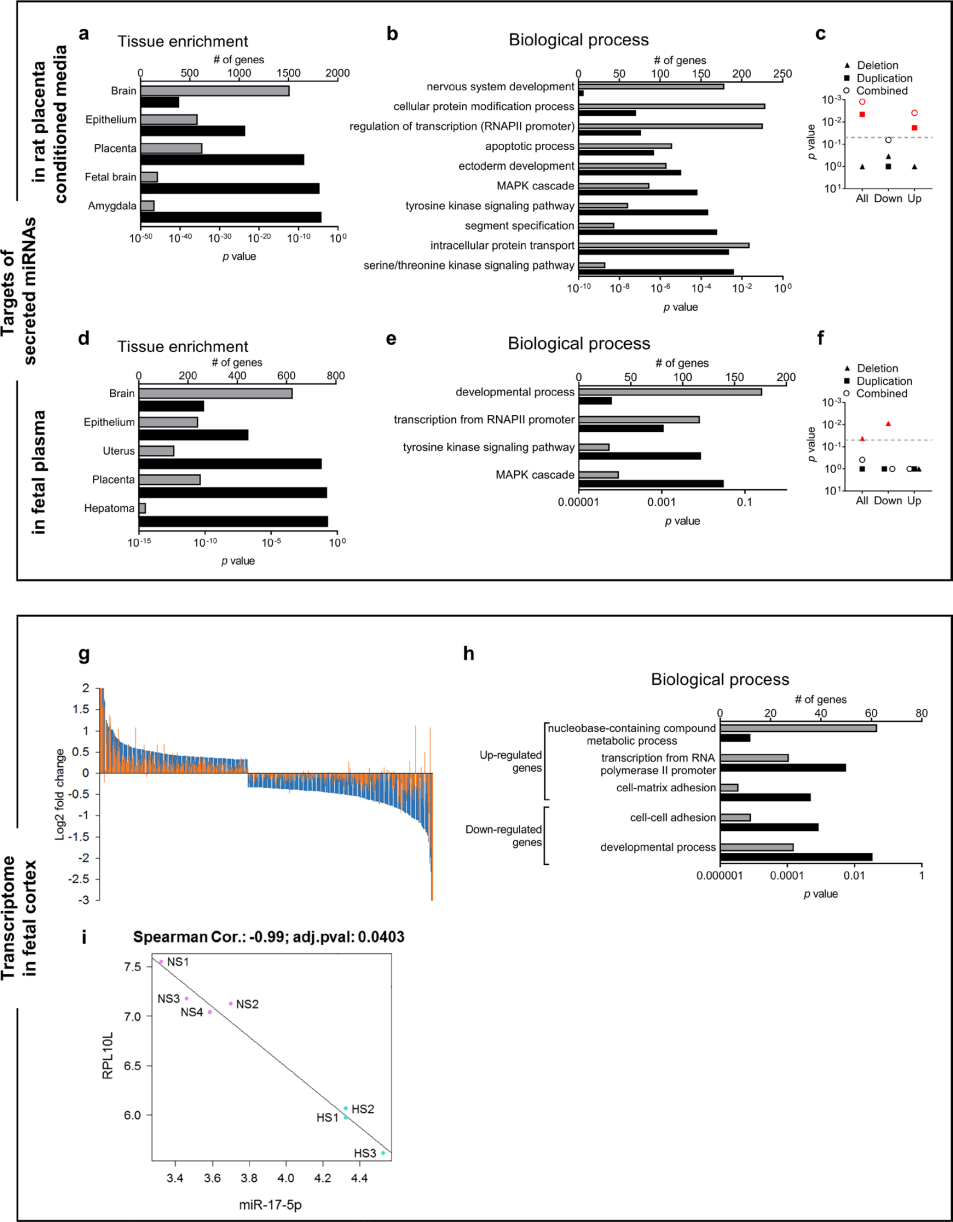
Characterisation of secreted miRNAs and transcriptome changes in the fetal brain. (**a–f**) Predicted targets of miRNAs that were significantly altered in response to maternal hypoxia in conditioned medium from rat placenta (**a–c**) or in fetal plasma (**d–f**) were analysed for enrichment of tissue types (**a**,**d**) and of biological processes (**b**,**e**). Predicted targets of all miRNAs, of upregulated miRNAs and of downregulated miRNAs were investigated for enrichment in CNVs associated with schizophrenia. Significant enrichment at *p* < 0.05 (grey line) shown in red (**c**,**f**). (**g–i**) Changes in the transcriptome were measured in the fetal cortex of offspring exposed to gestational normoxia or hypoxia and maternal administration of saline or MitoQ-NPs. Log2-fold changes of significant differentially expressed genes under hypoxia alone (blue) or hypoxia plus MitoQ-NP application (orange), relative to control levels (normoxia + saline) are shown (**g**). Significantly enriched biological processes of differentially expressed genes; the grey bars represent number of proteins assigned to each gene ontology term and the black bars show the respective *p* value (**h**). Correlation of miR-17-5p abundance changes in fetal plasma with expression changes of the gene coding for Ribosome protein L10-like (RPL10L) in fetal brain, under Normoxia + Saline (NS) and Hypoxia + Saline (HS) conditions (**i**). Log-transformed counts are shown. *p* value was adjusted for multiple comparisons using Benjamini-Hochberg.

Having identified factors, such as miRNAs, which were differentially secreted from the placental barrier following a hypoxic insult, we wanted to investigate if these had the potential to play a role in shaping the brain during fetal development. miRNAs regulate mRNA abundance^55^ and have been shown to be involved in neurodevelopment and disease^56^. Moreover exosomes, extracellular vesicles known to carry miRNAs, can cross the blood-brain barrier and reach the brain^57^. Using bioinformatics analyses we investigated the predicted target genes of those miRNAs that were differentially expressed in rat placenta conditioned media and in fetal plasma, in response to gestational hypoxia. The predicted target genes were enriched in the brain compared to other organs (p < 0.001) (Fig. 4a,d) as well as in biological processes linked to development (Fig. 4b,e). Furthermore, predicted miRNA targets were enriched within copy number variants (CNVs) found in schizophrenia cases compared to CNVs found in matched controls (Fig. 4c,f; Supplementary Table S5). An additional analysis conditioned on a minimal set of CNS-linked gene-sets previously shown to be enriched for schizophrenia CNVs in the same case-control data^58^; this indicated that while some of the enrichment seen in our predicted targets was shared with these known associated gene sets, a significant proportion seems to be independent (Supplementary Table S6). These results suggest that the differentially secreted miRNAs could target genes involved in neurodevelopment and neurodevelopmental disease.

Next we investigated if gene expression was altered in the fetal brain exposed to gestational hypoxia and if these changes may be correlated with the changes in secreted miRNAs. The mRNA levels of 510 genes were significantly altered in the fetal brain after gestational hypoxia. Maternal MitoQ-NP administration reduced these effects (Fig. 4g). Genes down-regulated following hypoxia were enriched for developmental processes (Fig. 4h). Differentially expressed genes in the fetal brain were significantly enriched for predicted targets of those miRNAs that had been found to be significantly altered in fetal plasma and conditioned medium (Supplementary Table S7), suggesting a potential link between secreted miRNAs and cortical transcriptome changes following a hypoxic insult. For a subset of significant miRNAs, including miR-17-5p (Fig. 4i), negative correlation with one or more significant mRNA changes in the brain was detected (Table 1).

**Table 1:** Significant correlations between miRNAs in fetal plasma and transcripts in fetal brain.

	mRNA	miRNA log2 FC	mRNA log2 FC	Correlation coefficient	Adjusted *p* value
rno-miR-1	HSPA5	−0.92	0.71	−0.992	0.036
	PDIA4	−0.92	0.64	−0.993	0.035
	XBP1	−0.92	0.46	−0.992	0.035
	RSPH1	−0.92	0.38	−0.994	0.035
rno-miR-17-5p	RPL10L	0.81	−1.29	−0.990	0.040
rno-miR-20a + rno-miR-20b-5p	ENG	−0.61	0.53	−0.989	0.042
	NOVA2	−0.61	0.36	−0.989	0.043
rno-miR-300-3p	HYOU1	−0.55	0.45	−1.000	0.035
	MGLL	−0.55	0.36	−0.990	0.036
	VASP	−0.55	0.33	−0.992	0.035
rno-miR-369-3p	FANCG	−0.58	0.66	−0.990	0.038
	FAM98A	−0.58	0.25	−0.994	0.035
	ENAH	−0.58	0.21	−0.991	0.035
rno-miR-410	XBP1	−0.50	0.46	−0.993	0.035
	RSPH1	−0.50	0.38	−0.992	0.035
rno-miR-451	EPHB4	−0.46	0.31	−0.988	0.049
rno-miR-499	NOVA2	−0.69	0.36	−0.991	0.036

miRNAs that were significantly altered in fetal plasma following gestational hypoxia were probed for negative correlation with mRNA that showed significantly changed levels in the fetal brain following the hypoxic insult. *p* values were adjusted for multiple comparisons using the Benjamini-Hochberg method.

The results indicate that maternal MitoQ-NP treatment can partially prevent hypoxia-induced gene expression changes in the fetal brain and point towards a potential link between miRNAs secreted from the placenta and the transcriptome of the developing brain.

### Neurodevelopmental effects of MitoQ-NPs *in vitro* and *in vivo*

The placenta may play an important role in the aetiology of neurodevelopmental disorders^21, 30–32^. Therefore, we further explored the potential link between placental secretions and changes in the offspring brain. We examined whether fetal plasma and media conditioned by BeWo barriers or rat placenta had similar biological effects on embryonic cortical neurons in culture. We further investigated if these effects might also be noted *in vivo* within the offspring brains at a later stage of development. We chose parameters that have relevance to psychological disorders^9, 17, 24, 59^.

**Figure 5:**
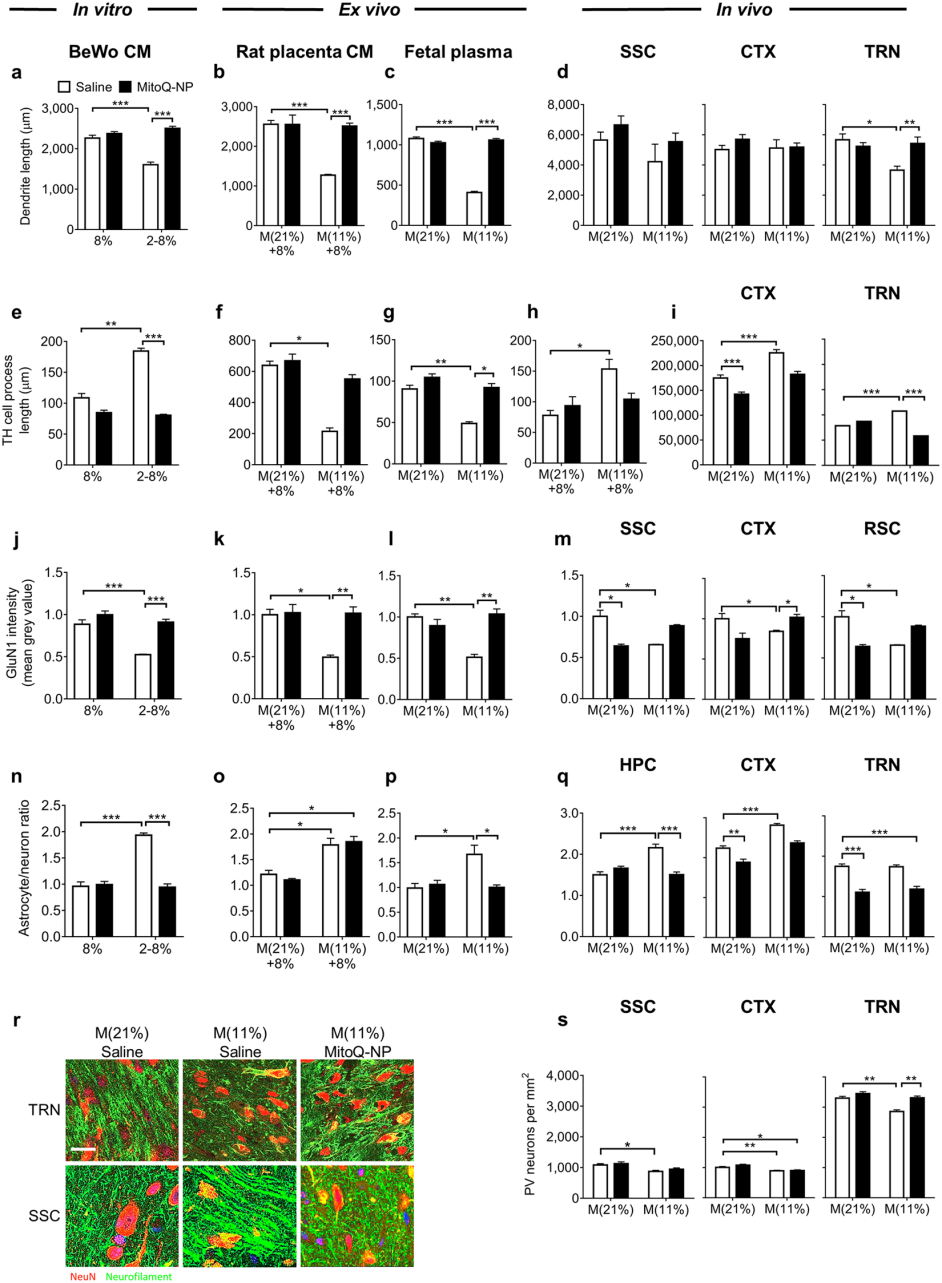
Effects of MitoQ-NPs on neurons. Effects of gestational hypoxia and MitoQ-NP treatment on neurons were examined *in vitro* by applying culture media conditioned by bilayered BeWo barriers or offspring placenta, or by applying fetal plasma to cortical cultures. Placental tissue and fetal plasma were collected from animals exposed to 6 d gestational normoxia, M(21%), or gestational hypoxia M(11%), in combination with maternal injection of either saline or MitoQ-NPs. Placental tissue was subsequently incubated *ex vivo* for 24 h under 8% oxygen conditions before collection of conditioned medium. BeWo barriers were cultured at 8% oxygen to mimic physiological oxygen conditions or at 2% oxygen followed by 8% oxygen to mimic hypoxia-rexoygenation (2–8%). (**a–d**) Dendrite lengths *in vitro* following exposure to BeWo conditioned medium (**a**; number of biological replicates from left to right: *n* = 25,25,15,15), placenta conditioned medium (**b**; *n* = 15,10,8,24) or plasma (**c**; *n* = 21,18,23,18) and *in vivo* (**d**) in somatosensory cortex (SSC; *n* = 8 different brains), combined somatosensory, auditory and retrosplenial cortex (CTX; *n* = 21,23,21,23) or thalamic reticular nucleus (TRN; *n* = 8). (**e–i**) Process lengths of tyrosine hydroxylase (TH)-positive neurons *in vitro* following exposure to BeWo conditioned medium (**e**; *n* = 25,25,15,15), placenta conditioned medium (**f**; *n* = 12,10,8,10), plasma (**g**; *n* = 21,27,19,19) or in neuron only-cultures following exposure to placenta conditioned medium (**h**; *n* = 5,5,5,5). (**i**), TH^+^ process length *in vivo* in CTX (*n* = 121,108,123,98) and TRN (*n* = 63,56,59,43). (**j–m**) GluN1 receptor subunit staining intensity *in vitro* following exposure to BeWo conditioned medium (**j**; *n* = 15,15,8,9), placenta conditioned medium (**k**; *n* = 10,10,15,10) or plasma (**l**; *n* = 28,10,17,18), and *in vivo* (m) in SSC (*n* = 3), CTX (*n* = 3) and retrosplenial cortex (RSC; *n* = 3). (**n–q**) Ratio of astrocytes to neurons *in vitro* following exposure to BeWo conditioned medium (**n**; *n* = 15,15,18,12), placenta conditioned medium (**o**; *n* = 10) or plasma (**p**; *n* = 5) and *in vivo* (**q**) in hippocampus (HPC, *n* = 23,18,18,24), CTX (*n* = 70,66,72,77) and TRN (*n* = 32,40,44,28). (**r**) Representative images of neurons in TRN and SSC. Sections were stained for neuronal marker NeuN (red) and somato-dendritic marker neurofilament (green). Scale bar = 25 μm. (**s**) Density of parvalbumin (PV)-positive neurons *in vivo* in SSC (*n* = 21,42,42,33), CTX (*n* = 63,126,126,98) and TRN (*n* = 35,51,55,43). *p < 0.05, **p < 0.01, ***p < 0.001, Tukey’s test following ANOVA.

In cortical cultures, exposure to conditioned media and plasma collected following a hypoxic episode caused a shortening of dendrites (Fig. 5a–c; Supplementary Fig. S5), a reduction of tyrosine hydroxylase (TH)-positive process lengths (Fig. 5f,g; Supplementary Fig. S5), a loss of GluN1 receptor subunit staining (Fig. 5j–l; Supplementary Fig. S5) and an increase in astrocyte-to-neuron ratio (Fig. 5n–p; Supplementary Fig. S5). In general, such changes were also seen in the brain (Fig. 5d,m,q). A decrease in dendrite length was restricted to the thalamic reticular nucleus (TRN; Fig. 5d) although thick, abnormally branched dendrites were noted in the somatosensory cortex (SSC; Fig. 5r). Process lengths of TH^+^ neurons were increased in cortex (CTX) and TRN (Fig. 5i), which contrasted with the reduction in process length observed after exposing cortical cultures to fetal plasma or rat placenta conditioned medium (Fig. 5f,g). However, the directionality of the effect observed *in vivo* was replicated when cortical cultures were exposed to BeWo conditioned medium (Fig. 5e) and when neuron-only cultures were exposed to media conditioned by rat placenta (Fig. 5h).

In general, the actions of MitoQ-NP treatment were also similar across the different models and either significantly prevented the effects of hypoxia, or caused hypoxia to no longer have significant effects, on dendrite length (Fig. 5a–d; Supplementary Fig. S5), TH^+^ cell process length (Fig. 5e–i), GluN1 receptor subunit staining (Fig. 5j–m; Supplementary Fig. S5) and astrocyte-to-neuron ratio (Fig. 5n–q). Additionally, the numerical density of parvalbumin-containing cells was significantly reduced in TRN, CTX and SSC of offspring following maternal hypoxia and rescued in the TRN by MitoQ-NP injection (Fig. 5s). Total cell count was not affected by either hypoxia or MitoQ-NP application *in vitro* or in the TRN, whereas a reduction in cell number was observed in the CTX (Supplementary Fig. S6). The number of TH^+^ cells were reduced in cortical cultures only after additional severe hypoxia was applied to the placenta *ex vivo* and no change was observed *in vivo* (Supplementary Fig. S6).

**Figure 6:**
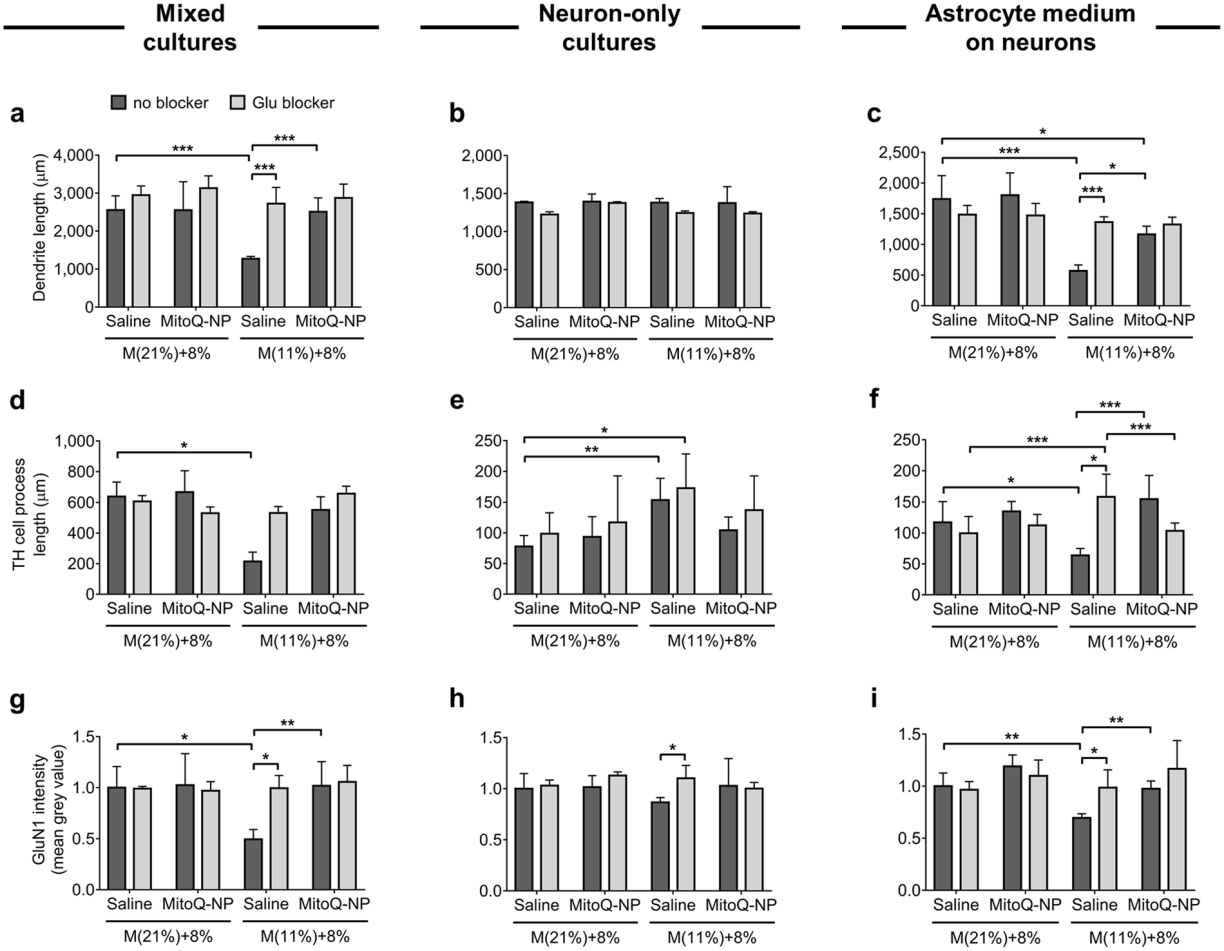
Roles of astrocytes and glutamate signalling in mediating the effects of maternal hypoxia and MitoQ-NPs on neurodevelopment. Mixed cortical cultures (**a**,**d**,**g**) or neuron-only cortical cultures (**b**,**e**,**h**) were incubated with rat placenta conditioned media. The latter were also incubated with medium conditioned by astrocytes which had previously been exposed to rat placenta conditioned media (**c**,**f**,**i**). Placentas were collected from pregnant rats that had been exposed to normoxia, M(21%), or hypoxia, M(11%), in combination with an injection of saline or MitoQ-NP. This was followed by incubation of the placenta in culture medium for 24 h *ex vivo* at 8% oxygen. To some of the cortical cultures NMDA receptor antagonist MK-801 was added with the conditioned medium. (**a–c**) Dendrite lengths of neurons (number of biological replicates from left to right: **a**, *n* = 5; **b**, *n* = 5, 4, 5, 3, 3, 3; **c**, *n* = 5; **d**, *n* = 10). (**d**–**f**) Dendrite lengths of tyrosine hydroxylase (TH)-positive neurons (*n* = 5, 3, 5, 3, 3, 3). (**g–i**) Staining intensity of GluN1 receptor subunits (**e**, *n* = 7, 9, 5, 7; **f**, *n* = 3; **g**, *n* = 5; **h**, *n* = 12, 10, 10, 11, 25, 10). Significant differences determined using Tukey’s test following ANOVA (*p < 0.05, **p < 0.01, ***p < 0.001).

Recently it was demonstrated that lipopolysaccharide, which shows minimal transport across the blood-brain barrier, may nonetheless have toxic actions within the brain by acting on astrocytes located at the blood-brain barrier^60^. We noted previously that glutamate may play a role in mediating the effects of hypoxia-conditioned media on cortical cultures^43^. We therefore tested the effects of media conditioned by rat placenta after maternal hypoxia on astrocyte signalling to neurons with and without glutamate blockade (Fig. 6). Dendrite lengths and GluN1 staining intensity were decreased in mixed astrocyte/neuron cultures (Fig. 6a,g), unchanged in neuron-only cultures (Fig. 6b,h) and decreased if astrocyte cultures were first exposed and then this astrocyte-conditioned medium was applied to neuron-only cultures (Fig. 6c,i). Moreover these effects were blocked by the NMDA receptor antagonist MK-801. In contrast, TH^+^ cell processes, although decreased in mixed cultures (Fig. 6d) and after exposure to the astrocyte-conditioned media (Fig. 6f), were increased in neuron-only cultures, an effect that was not altered by the glutamate antagonist (Fig. 6e).

Together these results suggest that maternal administration of MitoQ-NPs can ameliorate some of the long-term effects of gestational hypoxia on the offspring brain. These effects can be replicated in cortical cultures via exposure to rat placenta conditioned media or fetal plasma and may involve astrocyte-to-neuron signalling, partially via glutamate.

## Discussion

We showed that maternal injection of a single dose of NP-bound antioxidant MitoQ, during exposure to gestational hypoxia, reduced oxidative stress in the placenta and prevented a reduction of birth-weight (Supplementary Fig. S7). Birth-weight is a strong predictor for disease in later life^61^ and low birth-weight has been associated with altered neuropsychiatric outcomes^2^. Importantly the nanoparticle-bound drug appeared to exert its effect on the fetus indirectly, without passing through the placenta to the fetus. This is supported by the lack of nanoparticles detected in the fetus, the lack of MitoQ in conditioned media from BeWo barriers or rat placenta along with the lack of effect of drug treatment on oxidative stress in the fetal liver or brain compared to maternal liver and the placenta. As large NPs (180 nm) were used, these observations correlate with previous findings that the passage of intravenously injected NPs across the placenta depends on their size^62^. MitoQ-NP treatment was also found to reduce oxidative stress in the placenta of control animals, which had not been exposed to hypoxia, as well as in maternal tissues. The lack of gross changes in the dams and control fetuses in response to maternal MitoQ-NP injection suggests that the treatment did not have adverse effects. However, a more detailed assessment is required to comprehensively investigate if MitoQ-NP administration could adversely, or beneficially, affect maternal health or fetal health in pregnancies without placental oxidative stress.

Furthermore, we observed that maternal MitoQ-NP treatment prevented some abnormal changes in neurons in response to gestational hypoxia. Shortening of dendrites and a reduction in the density of immunostaining of NMDA receptor subunit GluN1 were detected in response to hypoxia. Similar types of change have been reported in neuropsychiatric disease such as schizophrenia or autism^13, 15, 17–22^. While shortening or lengthening of TH-positive processes has not been reported in neuropsychiatric disease, both schizophrenia and autism have been associated with increased tyrosine hydroxylase staining in substantia nigra^17^ as well as abnormal dopamine and dopaminergic receptor levels in substantia nigra, striatum and prefrontal cortex^17, 18^. The offspring brain in the presented study also showed a loss of parvalbumin-containing cells in cortex and reticular nucleus, which has been linked to schizophrenia and ASD-like behaviours^19, 20^, and a reduction in astrocyte/neuron ratio. Previous studies of neurodevelopmental disease have suggested that astrocyte number may be differently affected in different regions of the brain^23^. As it is known that males show higher vulnerability to several neurodevelopmental disorders, including schizophrenia and autism^63^, it would be of future interest to investigate differences in anatomical changes between male and female offspring brains following hypoxia and MitoQ-NP treatment and to establish if the treatment may be more beneficial in one sex over the other.

In addition to the neuroanatomical changes observed in offspring brains, changes in gene expression, among those genes involved in developmental processes, were detected within the fetal brain, in response to gestational hypoxia and MitoQ-NP treatment. Due to the lack of oxidative stress in the fetal brain following gestational hypoxia, the observed neuroanatomical and gene expression changes are less likely caused by a direct effect of the altered environmental oxygen and suggest an indirect effect of maternal hypoxia, potentially via the placenta. The observation that many of the morphological changes in the offspring brain were replicated *ex vivo* in cortical cultures exposed to culture medium conditioned by rat placenta or to fetal blood support the hypothesis that signalling from the placenta may play a role in neurodevelopment *in utero*. This is in keeping with previous findings showing that cortical cultures exposed to culture media conditioned by placenta following *ex vivo* hypoxia/reoxygenation, had shorter dendrites and altered levels of glutamate receptors^43^. The observation that the effects of fetal blood and conditioned media on neurons could be prevented by maternal treatment with MitoQ-NPs further supports an involvement of the placenta, as MitoQ-NPs were localised and exerted their biological effects on oxidative stress in the placenta and not in the fetus. Moreover, the damaging effects of placental secretions were prevented six days after maternal injection with MitoQ-NPs.

The effects on cortical cultures were also replicated following controlled hypoxic exposure in a simple *in vitro* barrier model consisting of cytotrophoblast cells, which in the human face the fetal circulation. This suggests that oxidative stress in the placental barrier may mediate effects on the offspring brain, as other factors, such as hormones that could be altered by the maternal hypoxia would not have been present in the *in vitro* model. We also observed a potential role of astrocytes in mediating the effects of gestational hypoxia and MitoQ-NP treatment. It would therefore be of interest to test whether molecules in fetal blood which were altered by gestational hypoxia could have actions in the brain across a fetal blood-brain barrier via astrocytic end feet.

Analysis of factors in fetal blood and secreted from the placenta *ex vivo*, showed that the miRNA profile was significantly altered by gestational hypoxia and partially ‘normalised’ by maternal injection of MitoQ-NPs. miRNAs, small RNAs that function as post-transcriptional regulators, have been shown to play an important role in neurodevelopment^56, 64^, brain function^65^ and psychiatric diseases^50, 56, 66^. Various circulating miRNAs are being studied in patient blood samples in order to develop biomarkers for psychiatric disease^66^. Schizophrenia has generally been associated with an increase in the miRNA biogenesis machinery and with a broad upregulation of cortical miRNAs^67^. Intriguingly, predicted targets of miRNAs significantly altered by gestational hypoxia in fetal blood and conditioned media were found to be enriched within CNVs associated with schizophrenia. Furthermore, a selection of miRNAs were negatively correlated with altered gene expression in the fetal brain. As an example, miR-17-5p was significantly upregulated following gestational hypoxia in both fetal blood and media conditioned by rat placenta; it was also significantly downregulated in response to maternal MitoQ-NP treatment. miR-17-5p upregulation was associated with the downregulation of ribosomal protein L10-like (RPL10L), a ribosomal-like protein with unknown function, in the fetal brain. The miR-17 family of miRNAs plays a role in early neurodevelopment by regulating cell proliferation and differentiation in the cortex^68^. Moreover, an increase in miR-17-5p levels in the human prefrontal cortex has been associated with schizophrenia^69, 70^. The results indicate that miRNAs secreted by the placenta could play a role in mediating the effects of gestational hypoxia and drug treatment on the developing fetus and suggest a tentative link between secreted miRNAs and gene expression in the fetal brain. While RNA-loaded extracellular vesicles have been shown to be able to cross the blood-brain barrier into the brain^57^ and extracellular miRNAs have been shown to be functionally active after uptake in target cells in the brain^71^, the hypothesis that placenta-secreted miRNAs may regulate gene expression in the fetal brain remains to be tested.

The proteomic profiles in fetal blood and conditioned media were also altered in response to gestational hypoxia and MitoQ-NP treatment, respectively. Especially lipoprotein-related proteins were found to be differentially abundant in foetal blood, which is of interest in view of the susceptibility of offspring to cardiovascular disease following gestational hypoxia^45, 72^. While an interplay between several different factors may be involved, the data strengthen the hypothesis of a mechanistic link between molecules secreted from the placenta, including miRNAs, and the effects of gestational hypoxia on the offspring brain. In some cases differential effects of hypoxia and drug treatment on the secretions in the fetal blood and in media conditioned by rat placenta were observed. A difference between the protein/miRNA profile of fetal blood and conditioned media is to be expected and this difference may reflect the complex interplay between molecules transported across the placenta, molecules secreted by the other fetal tissues, such as fetal liver, into fetal blood and molecules in the fetal blood that are regulated by the placenta. None of these factors would be present in the *ex vivo* preparation. In contrast, the protein/miRNA profile of culture medium conditioned by rat placenta would consist only of molecules released by the placenta, but in both directions, i.e. to the fetal and the maternal side.

The placenta has been shown to play a key role in fetal programming^21, 27–32^. The presented data suggest that treating the placenta with an antioxidant may prevent some changes in our models in response to hypoxia that are also considered to be important in the aetiology of human psychological disorders. Clinical trials with antioxidants in human disease have generally been disappointing, perhaps because not enough antioxidant reaches the mitochondria^73–77^. MitoQ has the advantage of being recycled back to its active form after detoxification of a free radical^78, 79^. Moreover, in our rat model MitoQ-NPs were able to produce long-lasting effects following administration of a single controlled dose whilst only affecting the placenta and not the fetus. We propose a future possibility of treating the placenta, and not the fetus, in order to reduce the potential for disease in later life.

## Methods

All animal procedures were conducted in accordance with the United Kingdom Animals Scientific Procedures Act (1986) following institutional ethical approval at the University of Birmingham and then carried out under Home Office project licence authority. Procedures conducted in Canada were approved by the University of Alberta Health Sciences Animal Policy and Welfare Committee, in accordance with the Canadian Council on Animal Care guidelines. All efforts were made to minimize any suffering and the number of animals used. All methods were performed in accordance with the relevant guidelines and regulation.

### Nanoparticles

The production of drug-loaded γ-PGA-Phe NPs^80^ is described in Supplemental Methods. Both *in vitro* and *in vivo* experiments used NPs carrying a final dose of 0.5 μM MitoQ.

### Cell cultures

Bilayered cell barriers were prepared using BeWo cells, a choriocarcinoma cell line (gift from Dr Margaret Saunders, University of Bristol, UK)^40, 41^ grown on Transwell 0.4 μm pore polyester inserts (Corning, UK). Culture media for BeWo barriers consisted of Dubecco’s Modified Eagle’s Medium (DMEM) with F-12 (Sigma-Aldrich) containing 10% FBS (Thermo Fisher Scientific) and 2 mM L-glutamine (Sigma-Aldrich). Cell lines were routinely tested for mycoplasma contamination. Integrity of cell barriers was tested by applying fluorescein isothiocyanate-conjugated BSA (FITC-BSA; Sigma-Aldrich) at a final concentration of 100 μg/mL to the top of cell barriers in Transwell inserts and an equal concentration of non-conjugated BSA to the medium below barriers. Fluorescence intensity was measured 1 h later in the media below the barriers and in the insert.

Cortical cultures were prepared from dissociated rat E18 cortical tissue and grown on glass coverslips as described previously^43^, in Gibco Neurobasal media with 1x Gibco B-27 Supplement, 1x antibiotic-antimycotic (all Thermo Fisher Scientific) and 2 mM L-glutamine. To produce neuron-only cultures, 40 μM 5-fluoro-2-deoxyuridine was added to each well at 5 days *in vitro*. Astrocyte-only cultures were prepared by growing cortical cultures in DMEM with 10% FBS, 2 mM L-glutamine and 1x antibiotic-antimycotic. Cultures were accepted as astrocyte-only if neuron number was zero in three representative coverslips. The effect of glutamate was investigated by adding MK-801, a NMDA receptor antagonist, at a concentration of 10 μM to each well and incubating for 4 h before exposure of the cells to conditioned media. Exposures of cortical cultures were performed at a minimum in triplicate for condition medium from each of 3 placenta (one from each dam), creating a minimum of 9 data points for each condition. At least three different sets of cortical cultures were tested.

### Conditioned media

Conditioned tissue culture media were prepared using bilayered barriers described above or using rat placentas isolated from the *in vivo* experiment (see below). Barriers were grown and tissues were incubated at 21%, 8% or 2% oxygen in a SCI-tive hypoxia chamber (Baker Ruskinn, USA). This was followed by a 24 h exposure period, at the start of which the media under the barriers or around the tissue was replaced. Some wells were additionally exposed to NPs above the barrier. The media was then conditioned by the explant or barrier for 24 h at the same or altered oxygenation. Conditioned media were applied to embryonic cortex for 6 days from day 12 *in vitro* to test their effects on cell number, dendrite length, tyrosine hydroxylase positive cell processes and glutamate receptors as previously described^40, 41^. Cortical cultures were also exposed to conditioned medium collected from astrocyte-only cultures that had been exposed for 24 h to rat placenta-conditioned medium. Potential leakage of MitoQ from barriers or placenta was measured in the conditioned medium using liquid chromatography-tandem mass spectrometry (LC-MS/MS)^81^.

### Immunocytochemistry

Cortical cultures were fixed in supercold methanol (−20 °C), washed with PBS and blocked with 5% BSA, 5% NGS in PBS for 30 min. They were incubated with primary antibodies against MAP2 (1:2000, #188004; Synaptic Systems, Germany), GFAP (1:1000, #3670; Cell Signalling Technology), tyrosine hydroxylase (1:500, ab112; abcam) and GluN1 (1:500, ab9864; Merck Millipore), overnight at 4 °C. Sections were stained with secondary antibodies Alexa Fluor 488 anti-rabbit IgG, Alexa Fluor 488 anti-mouse IgG or Alexa Fluor 488 anti-guinea pig IgG (all Thermo Fisher Scientific, diluted 1:500) for 2 h at room temperature under minimal light conditions, washed with PBS and mounted in DAPI mounting media. Five images per coverslip were taken on a confocal microscope (SP2-AOBS, Leica). Analysis of the slides was performed with the experimenter blind to the experimental group. Dendrite lengths were measured using ImageJ. For receptors images were taken at x64 (with oil) on a fluorescence microscope (Leica SP5II) after excitation at 488 nm. Using ImageJ, images were converted to RGB files then measurements were taken of the mean grey value of each image providing an average of the relative intensity of the staining. Measurements were verified by cell counts based on DAPI staining. Background levels of fluorescence were ascertained in the absence of cells, primary antibody and/or secondary antibody.

### *In vivo* experiments

Three-month-old female Sprague-Dawley rats (Charles River, Wilmington, MA) were maintained on *ad libitum* standard rat chow and tap water in a 12:12-h light-dark cycle and acclimatized before breeding. Day 0 of pregnancy was determined by sperm in a vaginal smear. On gestational day (GD) 15 of pregnancy the rats were injected intravenously with saline (vehicle control) or 125 μM MitoQ-NPs and exposed for the next 6 days to 21% or 11% oxygen in an A-Chamber (BioSpherix, USA)^44^. Some rats were sacrificed at GD20. EDTA plasma collected from fetuses by decapitation was pooled per litter and flash frozen; placenta, maternal and fetal tissues were collected fresh, flash-frozen or fixed in formal saline. Other rats were allowed to give birth in normal oxygen conditions. Birth-weights were measured and offspring brains examined at postnatal day (P) 30.

Statistical tests were not used to predetermine sample size. Experimental groups were allocated according to a preassigned schedule, depending on the order in which successful pregnancy was established. From each litter two males and two females were randomly selected for examination at P30. For subsequent immunohistochemistry analysis of rat tissue, the experimenter was blinded to group allocation.

### Localisation of NPs

Pregnant rats were injected with 125 μM NPs conjugated with Alexa Fluor 488 and culled at GD16 to collect flash-frozen fetal, maternal and placental tissue. Sagittal sections of placental labyrinth and junctional zone, sections of fetal and maternal cortex and liver were produced using a cryostat and viewed with confocal microscopy for the localisation of fluorescent NPs.

### Analysis of placental, fetal and maternal tissues

Fixed placentas were processed for paraffin wax histology. Sections were cut at 3 μm and stained with haematoxylin and eosin and at 2 μm for avidin-biotin immunocytochemistry for CD34 (ab81289; abcam, UK) using a full automated Bond3 immunostaining machine with bond polymer refined detection (Leica) to ensure the same immunostaining method was applied to all sections. Images were captured on a fluorescent microscope (Leica SP5II) and analysed with ImageJ software. Numbers of biological replicates are given in figure legends. For measurements pertaining to placenta vasculature, values for each biological replicate are averages of technical replicates taken from multiple fields of view.

Levels of reactive oxygen species (ROS) were measured in fetal, maternal and placental tissues using the 2′,7′-dichlorofluorescein diacetate (DCFDA) assay. Sagittal sections (10 μm) were cut on the cryostat and exposed to 20 μM DCFDA solution in HBSS at 37 °C in a humidifying chamber. After counter-staining with DAPI, slides were immediately imaged using a confocal microscope (excitation and emission wavelengths of 495 nm and 529 nm, respectively). Using Image-Pro Premier 9.2 (Media Cybernetics, USA), fluorescence levels of DCF, the product of DCFDA deacetylation by cellular esterases and oxidisation by ROS, were quantified in labyrinth and junctional zone of the placenta, maternal and fetal liver, cortex and cerebellum.

### Analysis of conditioned media and plasma

BMP4, 6 and 9 were measured in conditioned media using a cell based assay^82^. Amino acids were measured as previously described^43^.

Total RNA was extracted from 200 μl conditioned media or 100 μl fetal plasma using the miRNeasy Mini Kit and the miRNeasy Serum/Plasma Kit (Qiagen, Germany). Small RNA and miRNA levels were measured using the Small RNA Kit on the 2100 Bioanalyzer (Agilent Technologies) at the University of Bristol Genomics Facility. Levels of individual miRNAs were analysed using the nCounter Rat v1 miRNA Expression Assay or the nCounter Human v2 miRNA Expression Assay (NanoString Technologies, USA), which detects 423 or 800 different species-specific miRNAs, respectively. Briefly, 3 μl of each undiluted sample were hybridised with barcoded probes and immobilised on an nCounter Cartridge. Barcode signals were counted using the nCounter Digital Analyzer.

Proteomic analysis was performed by the University of Bristol Proteomics Facility (see Supplementary Methods for details). Briefly, samples were depleted of rat albumin, digested with trypsin and labelled with Tandem Mass Tag (TMT) 10Plex reagents (Thermo Fisher Scientific). The labelled samples were fractionated by high pH reversed-phase chromatography followed by nano LC-MS/MS. The raw data files were processed and quantified using Proteome Discoverer software v1.4 (Thermo Fisher Scientific) and searched against the UniProt Rat database using the SEQUEST algorithm. All peptide data was filtered to satisfy a false discovery rate of 5%.

### RNA sequencing

RNA was extracted from 50 mg of fetal frontal cortex tissue at GD20 using the RNeasy Mini kit (Qiagen). RNA quality and integrity was measured on the 2100 Bioanalyzer. mRNA sequencing was performed by Edinburgh Genomics. Libraries were prepared from total RNA samples using the Illumina TruSeq stranded mRNA Sample Preparation Kit. Briefly the polyA RNA from 1 ug of total RNA was captured onto Oligo d(T) beads, before fragmentation and elution of the polyA RNA. The RNA was reverse transcribed using random primers and the resulting cDNA was double stranded. The cDNAs were ligated with adapters containing unique barcodes for each sample. Libraries were then assessed for size by electrophoresis and quantified by qPCR. The libraries were sequenced by 75 bases paired-end sequencing across two lanes of an Illumina HiSeq 4000. This level of sequencing produced greater than 34 million paired reads per sample. The FASTQ files were generated using the standard Illumina pipeline for bcl2fastq.

### Bioinformatic analyses

Differential expression analysis of NanoString miRNA data is detailed in Supplementary Methods. TargetScanHuman v7.0^83^ was used to create a list of potential (human-equivalent) targets of the significant miRNAs (Total Context Score < −0.2). Predicted target genes were analysed for pathway enrichment of CNVs associated with schizophrenia, as described by Pocklington *et al*.^58^. The analysis was applied to the combined ISC + MGS + CLOZUK dataset, comprising a total of 11,355 cases and 16,416 controls. The analyses are based on large, rare CNVs (>100 kb, frequency <1%), as these are both the most robustly called and most enriched in people with schizophrenia. The primary analysis was performed on all CNVs, with secondary analyses performed for deletions and duplications separately. A further analysis was performed conditioning on the ‘minimal’ gene sets previously shown to capture CNS-related gene set enrichment in this CNV dataset^58^.

Peptide spectra from the proteomics data set were analysed using moderated *t*-statistics from the empirical Bayes method to calculate differential protein abundance. Modified scripts, published by Kammers *et al*.^84^ and based on the limma package^85^, were used.

We used Tophat^86^ to align RNA sequencing reads to the rat reference genome Rnor_6.0 (GenBank Assembly ID GCA_000001895.4), and HTSeq^87^ to generate read counts from the resulting BAM files. Out of 672 million paired-end reads, nearly 557 million (82.8%) concordant pairs mapped to unique locations in the Rn6 version of the genome. Genes were analysed for differential expression and differentially expressed genes were further analysed for interactions with the miRNA data sets (see Supplementary Methods).

Differentially abundant proteins, differentially expressed genes and predicted targets of differentially secreted miRNAs were analysed for enrichment of biological processes, cellular compartments and tissues using the GO-slim feature in PANTHER 11.0^88, 89^ and the GO Direct and UP_TISSUE features in DAVID 6.8^90, 91^. Venn diagrams were produced using Venny 2.1 (http://bioinfogp.cnb.csic.es/tools/venny/index.html).

### Data availability

Raw and processed data files generated from NanoString and RNA sequencing analyses have been deposited in NCBI’s Gene Expression Omnibus^92^ and are accessible through GEO Series accession number GSE86410. The MS proteomics data have been deposited to the ProteomeXchange Consortium via the PRIDE partner repository^93^ with the dataset identifiers PXD004955 and PXD004956 for fetal plasma and conditioned medium, respectively.

### Immunohistochemistry of P30 brains

At P30 rats were anaesthetised with 4% isoflurane and perfused with 4% PFA. Brains were post-fixed in 4% PFA, placed in 30% sucrose until sunk and embedded in OCT. Three brains per condition were randomly selected, one from each litter. 12-μm cryostat sections were produced and mounted as contiguous triplicates. Brain sections were exposed to cold methanol at −20 °C for 10 min for fixation. For GluN1 staining, sections were fixed in 2% PFA, followed by permeabilisation in 0.3% Triton X-100 in PBS for 15 min. All slides were washed in PBS after every incubation step. Slides were blocked with 5% goat serum (Sigma-Aldrich), 0.3% Triton X-100 in PBS for 2 h at 4 °C, followed by an overnight incubation (or 48 h incubation for GluN1 staining) at 4 °C in primary antibody in PBS with 1% BSA, 0.3% Triton X-100. Antibodies used were raised against MAP2 (1:500, ab32454; abcam), NeuN (1:1000, ab177487; abcam), neurofilament (1:500, ab24575; abcam), GFAP (1:500, #3670; Cell Signaling Technology), parvalbumin (1:500, ab11427; abcam), tyrosine hydroxylase (1:500, ab112; abcam) and GluN1 (1:200, MAB1570, Merck Millipore). Slides were incubated with secondary antibody Alexa Fluor 555 anti-rabbit IgG, Alexa Fluor 488 anti-mouse IgG or Alexa Fluor 568 anti-mouse IgG (Thermo Fisher Scientific) at 1:500 for 2 h at 4 °C. Vectashield Mounting Medium with DAPI (Vector Laboratories, USA) was used to mount coverslips. Coronal sections were chosen to show thalamic reticular nucleus, primary somatosensory, primary auditory and retrosplenial granular cortex^94^. Damaged sections, due to problems with freezing or storage of the samples, were excluded from analysis after visual confirmation. Analysis of brain sections was done with the experimenter blind to the *in vivo* exposure. For each site, in each brain, a minimum of 5 fields of view were examined for each of 3 sections in both hemispheres using a LASX (Leica) widefield microscope or a SP5II (Leica) confocal microscope at x40 magnification using oil. This resulted in a minimum of 30 fields of view being analysed for each site. The analysis of the intensity of the IHC staining of GluN1 was determined using a similar approach to that used previously^95^. Briefly, nine sections per condition were imaged and 10–12 images were collected for every brain region per case and using the same microscopy settings of intensity and magnification to allow for a meaningful comparison. Photos were sorted and converted to greyscale then the total pixel number was determined using a corrected macro for ImageJ. The total pixel number was subsequently subtracted from the background.

### Statistics

Data are presented as means ± s.e.m. For all statistical comparisons, variances were similar in magnitude between the compared groups. As indicated in the figure legends, one-way or two-way ANOVA was performed in Prism 6.0 (GraphPad, USA) or SPSS 21.0 (IBM Corp., USA) with *post*-*hoc* analysis using Bonferroni correction or Tukey’s test for multiple comparisons. Two-way ANOVA was used to test for main effects of drug and of oxygen conditions and for interaction effects. For GluN1 staining intensity analysis, normality of data distribution was tested using the D’Agostino-Omnibus normality test in Prism 6.0 (*p* > 0.05) and parametric testing was applied to test for differences of means between groups using multiple unpaired, two-sided Student’s *t*-test *post*-*hoc* testing adjusted for multiple comparisons using Bonferroni correction.

## Electronic supplementary material

Supplementary Information
