# Supplementary material for: Treating the placenta to prevent adverse effects of gestational hypoxia on fetal brain development
Source: Sci Rep. 2017 Aug 22;7:9079. doi: 10.1038/s41598-017-06300-1 (PMC5567270; doi:10.1038/s41598-017-06300-1)
Supplement: Supplementary file 1 — Supplementary Information [file 41598_2017_6300_MOESM1_ESM.pdf]

SUPPLEMENTARY INFORMATION

Treating the placenta to prevent adverse effects of gestational hypoxia on fetal brain development

Tom J Phillips*, Hannah Scott*, David A Menassa, Ashleigh L Bignell, Aman Sood, Jude S Morton, Takami Akagi, Koki Azuma, Mark F Rogers, Catherine E Gilmore, Gareth J Inman, Simon Grant, Yealin Chung, Mais M Aljunaidy, Christy-Lynn Cooke, Bruno R Steinkraus, Andrew Pocklington, Angela Logan, Gavin P Collett, Helena Kemp, Peter A Holmans, Michael P Murphy, Tudor A Fulga, Andrew M Coney, Mitsuru Akashi, Sandra T Davidge, C Patrick Case#

Supplementary Data

Production of drug-bound nanoparticles

The drugs Gap26, MitoQ, PPADS and C17 were adsorbed by hydrophobic and electrostatic interaction with NPs consisting of an amphiphilic copolymer of poly(γ-glutamic acid) and L-phenylalanine ethylester (γ-PGA-Phe NPs). The adsorption efficiency of the drug was 30-45% (Supplementary Fig 1a), with the amount adsorbed depending on its initial concentration (Supplementary Fig 1b,c). The sizes of Gap26 (50 μg/1mg NP), MitoQ (45 μg/1mg NP) and C17 (20 μg/1mg NP)-adsorbed NPs were 260 nm (polydispersity index PDI = 0.14), 180 nm (PDI = 0.09) and 220 nm (PDI = 0.08), respectively. The drug-adsorbed NPs showed a negative zeta potential (-20 mV) in PBS possibly due to the ionized carboxyl groups of γ-PGA on the NP surfaces. The NPs were stable under physiological conditions, exhibiting no aggregation, precipitation, or dissociation for at least 6 weeks. We observed a sustained release of the drug from NPs into PBS and tissue culture medium containing serum (Supplementary Fig 1d,e).

Effects of drug-bound nanoparticles on signalling through barriers in vitro

We investigated whether NP-bound drugs, like the drugs in their soluble form, would be able to prevent damaging secretions from bilayered BeWo barriers, a model of trophoblast barriers, in response to adverse stimuli. BeWo barriers were exposed *in vitro* to altered oxygenation, as a stimulus that would be relevant to fetal development, with and without administration of different drug-bound NPs. We tested the effects of culture media conditioned by BeWo barriers on fibroblasts (Supplementary Fig S2a,b) and dissociated neurons from embryonic cerebral cortex (Supplementary Fig S2c). As noted previously[40,41,43], the conditioned medium caused DNA damage in fibroblasts and dendrite shortening in neurons. Both types of damage have relevance to the biology of psychological disorders[96-98]. In all cases the damage was almost completely prevented by applying MitoQ-NPs to the barriers or explants at the start of the hypoxia/reoxygenation exposure (Supplementary Fig S2a-c). Gap26-NPs were also effective in preventing damage to fibroblasts and neurons mediated by indirect hypoxia-reoxygenation (Supplementary Fig S2a-c). Both NP-bound MitoQ and Gap26 were as effective as the soluble drugs (Supplementary Fig S2b)[40,41]. Unloaded NPs were ineffective (Supplementary Fig S2b). Dendrite shortening was also prevented by MitoQ-NPs and Gap 26-NPs when BeWo barriers were exposed to the toxic benzene metabolites benzoquinone and hydroquinone (Supplementary Fig S2d). Again unloaded NPs were ineffective. NPs bound to PPADS were least effective at preventing cell damage (Supplementary Fig S2a,c).

To confirm that the NPs did not cause these changes by passing through the barriers, we exposed cortical cells (Supplementary Fig S2e-h) or fibroblasts (Supplementary Fig S2i,j) directly to NPs. Direct application of MitoQ-NPs, unloaded NPs or MitoQ alone did not affect dendrite lengths (Supplementary Fig S2e), cell number (Supplementary Fig S2f),

number of neurons (Supplementary Fig S2g) or number of astrocytes (Supplementary Fig S2h). Furthermore, MitoQ-NPs or unloaded NPs had no direct effect on cell viability of fibroblast (Supplementary Fig S2i). Neither MitoQ-NPs nor Gap26-NPs affected DNA integrity in fibroblasts (Supplementary Fig S2j). Direct application of Gap26-NPs, but not MitoQ-NPs, reduced the amount of DNA damage in fibroblasts caused by direct exposure to benzoquinone and hydroquinone (Supplementary Fig S2j), suggesting that some of the DNA damage might be caused by bystander signalling through connexin channels within the fibroblast cultures[42]. Furthermore, the BeWo barriers showed high integrity (Supplementary Fig S2k), confirming that NP-bound drugs were unable to pass through gaps between the cells to directly affect fibroblast and cortical cultures.

Supplementary Methods

Nanoparticles

An amphiphilic copolymer of poly(γ-glutamic acid) and L-phenylalanine ethylester(γ-PGA-Phe) was synthesized as described previously[80] using a 50% Phe grafting degree. 10 mg/mL of γ-PGA-Phe was dissolved in DMSO, added to equivalent volume of 0.15 M NaCl and dialyzed against distilled water. Dialyzed solutions were freeze-dried and resuspended in PBS (10 mg/mL). NPs were measured by dynamic light scattering (Zetasizer Nano ZS, Malvern Instruments, UK) as 180 nm diameter, polydispersity index 0.12, Zeta potential -20 mV.

γ-PGA-Phe NPs (10 mg/mL) were mixed with Gap26 (2 mg/mL), MitoQ (2 mg/mL) or C17 (0.5 mg/mL) at equivalent volume in 0.2 M NaCl, and incubated at 4°C for 12 h. NPs were isolated by centrifugation, washed and resuspended in PBS to 10 mg/mL. The amount of drug which was adsorbed to NPs was evaluated by the Lowry method for Gap26, and UV absorption measurement for MitoQ (278 nm) and C17 (331 nm). The adsorption efficiency (%) was calculated as (adsorbed drug weight/initial feeding weight) × 100. Drug loading was calculated as loaded drug weight (μg)/NP weight (mg). Release of drug from NPs *in vitro* was calculated by immersing them in PBS or culture medium with 10% FCS and measuring the drug in the supernatant.

Measurement of cell viability and DNA damage in fibroblasts

Primary human BJ skin fibroblasts (ATCC CRL-2522) were grown in Minimal Essential Medium (Sigma-Aldrich), supplemented with 10% FBS, 2% HEPES buffer (Sigma-Aldrich), 1 mM sodium pyruvate solution (Thermo Fisher Scientific), 100 U/mL penicillin-streptomycin and 2 mM L-glutamine. Conditioned media from BeWo barriers exposed to hypoxia-reoxygenation or to 30 μM hydroquinone and 30 μM benzoquinone with and without NPs for 24 h were applied to fibroblasts to assess cell viability by Trypan Blue assay

and DNA damage by alkaline comet assay, as described previously[40,41]. The DNA damage was measured using the mean tail moment as defined by the product of comet length and tail intensity.

<u>Nanoparticle Tracking Analysis</u>

Extracellular vesicles were extracted from conditioned medium using the Total Exosome Isolation Reagent (Thermo Fisher Scientific), according to manufacturer's instructions, and resuspended in 100 µl PBS. Samples were measured using the Nanosight NS500 NTA instrument (Malvern Instruments, UK). Ten consecutive 30-second recordings were made for each sample and processed using the NTA 2.3 software (Nanosight Ltd.). Prior to analysis, the instrument was calibrated using reference silica and polystyrene nanospheres.

<u>Proteomic analysis</u>

TMT Labelling and high pH reversed-phase chromatography

Experimental samples were depleted of albumin using a Pierce Albumin Depletion Kit (following the protocol for rat albumin). For the fetal plasma samples, a pooled 'standard' was then generated by combining an equal aliquot (6 µg) of each of these albumin-depleted samples. 40 µg of each experimental sample or the pooled standard was then digested with trypsin (2.5 µg trypsin per 100 µg protein; 37°C, overnight) and labelled with Tandem Mass Tag (TMT) 10plex reagents according to the manufacturer's protocol (Thermo Fisher Scientific, Loughborough, UK) and the labelled samples were pooled.

An aliquot of the pooled sample was evaporated to dryness and resuspended in buffer A (20 mM ammonium hydroxide, pH 10) prior to fractionation by high pH reversed-phase chromatography using an Ultimate 3000 liquid chromatography system (Thermo Fisher

Scientific). In brief, the sample was loaded onto an XBridge BEH C18 Column (130 Å, 3.5 µm, 2.1 mm x 150 mm, Waters, UK) in buffer A and peptides eluted with an increasing gradient of buffer B (20 mM Ammonium Hydroxide in acetonitrile, pH 10) from 0-95% over 60 minutes. The resulting fractions were evaporated to dryness and resuspended in 1% formic acid prior to analysis by nano-LC MS/MS using an Orbitrap Fusion Tribrid mass spectrometer (Thermo Scientific).

Nano-LC Mass Spectrometry

High pH RP fractions were further fractionated using an Ultimate 3000 nanoHPLC system in line with an Orbitrap Fusion Tribrid mass spectrometer (Thermo Scientific). In brief, peptides in 1% (vol/vol) formic acid were injected onto an Acclaim PepMap C18 nano-trap column (Thermo Scientific). After washing with 0.5% (vol/vol) acetonitrile 0.1% (vol/vol) formic acid peptides were resolved on a 250 mm × 75 µm Acclaim PepMap C18 reverse phase analytical column (Thermo Scientific) over a 150 min organic gradient, using 7 gradient segments (1-6% solvent B over 1 min, 6-15% B over 58 min, 15-32% B over 58 min, 32-40% B over 5 min., 40-90% B over 1 min, held at 90% B for 6 min and then reduced to 1% B over 1 min) with a flow rate of 300 nL min^{-1}. Solvent A was 0.1% formic acid and Solvent B was aqueous 80% acetonitrile in 0.1% formic acid. Peptides were ionized by nano-electrospray ionization at 2.0 kV using a stainless steel emitter with an internal diameter of 30 µm (Thermo Scientific) and a capillary temperature of 275°C.

All spectra were acquired using an Orbitrap Fusion Tribrid mass spectrometer controlled by Xcalibur 2.0 software (Thermo Scientific) and operated in data-dependent acquisition mode using an SPS-MS3 workflow. FTMS1 spectra were collected at a resolution of 120,000, with an automatic gain control (AGC) target of 200,000 and a max injection time of 50 ms. The Top N most intense ions were selected for MS/MS. Precursors were filtered according to charge state (to include charge states 2-7) and with monoisotopic precursor selection.

Previously interrogated precursors were excluded using a dynamic window (40 s +/-10 ppm). The MS2 precursors were isolated with a quadrupole mass filter set to a width of 1.2 m/z. ITMS2 spectra were collected with an AGC target of 5,000, max injection time of 120 ms and CID collision energy of 35%.

For FTMS3 analysis, the Orbitrap was operated at 60,000 resolution with an AGC target of 50,000 and a max injection time of 120 ms. Precursors were fragmented by high energy collision dissociation (HCD) at a normalised collision energy of 55% to ensure maximal TMT reporter ion yield. Synchronous Precursor Selection (SPS) was enabled to include up to 5 MS2 fragment ions in the FTMS3 scan.

Data Analysis

The raw data files were processed and quantified using Proteome Discoverer software v1.4 (Thermo Scientific) and searched against the UniProt Rat database using the SEQUEST algorithm. Peptide precursor mass tolerance was set at 10 ppm, and MS/MS tolerance was set at 0.6 Da. Search criteria included oxidation of methionine (+15.9949) as a variable modification and carbamidomethylation of cysteine (+57.0214) and the addition of the TMT mass tag (+229.163) to peptide N-termini and lysine as fixed modifications. Searches were performed with full tryptic digestion and a maximum of 1 missed cleavage was allowed. The reverse database search option was enabled and all peptide data was filtered to satisfy false discovery rate (FDR) of 5%.

<u>Bioinformatics analyses</u>

Analysis of NanoString data

NanoString nCounter data consist of discrete sequence counts as a measure of miRNA expression within each sample. These counts are similar to counts from other high-

throughput methods such as RNA sequencing, where discrete statistical models such as the Poisson or negative binomial distributions may be used to estimate differences between samples[99,100]. Consequently, differential expression prediction methods originally designed for RNA sequencing data may also be used with NanoString data[101]. Following the procedures outlined in[101], we developed the following pipeline to assess DE-miRNA between samples: First, we converted each raw NanoString output file into a list of counts. Next we merged the counts for the replicates in each sample into a table with one column per replicate. To compare two samples, we merged the corresponding tables into a single table that we then passed into our differential expression analysis pipeline.

Differential expression analysis of NanoString and RNA sequencing data

RUVSeq was used to remove unwanted variation[102] and then edgeR[100] to predict differentially-expressed RNAs (miRNAs in the case of NanoString data, mRNAs in the case of RNA sequencing data). We selected edgeR for our pipeline as it may be more sensitive than DESeq[100,103,104] and it facilitates the use of RUVSeq[102]. We used RUVSeq to eliminate, as far as possible, variation from sources unrelated to the treatment groups, such as differences in blood plasma collection, centrifugation, enrichment, and RNA purification. We expected no differential expression between replicates within a treatment group and few differentially-expressed RNAs between treatment groups, so the relative log-expression should be consistent across all samples. In addition, the largest component of variation in the data should reflect RNAs that are differentially expressed between treatment groups. For each comparison, we first used RUVSeq to adjust the counts to account for unwanted variation, then used the adjusted counts with edgeR's generalized linear model to yield the final predictions.

To mitigate possible false-positives, miRNAs were classed as significant differentially secreted miRNAs if $p < 0.05$, if count ≥ 10 for at least one of the compared conditions

(except for fetal plasma miRNAs were counts were generally very low) and if there was an up or down regulation of at least 25%.

Analysis of miRNA-mRNA correlation

Enrichment analysis of the RNA sequencing data for predicted targets (derived from TargetScanHuman[83]) of significant miRNAs was performed in R/Bioconductor using the Fisher's exact test and investigating enrichment only. Correlation of abundance changes of significant miRNAs with abundance changes of significant mRNAs was analysed with the miRComb[105] package for R/Bioconductor, using Spearman correlation and Benjamini-Hochberg adjustment for multiple comparisons.

Supplementary References

96 Sertan Copoglu, U. *et al.* Increased oxidative stress and oxidative DNA damage in non-remission schizophrenia patients. *Psychiatry Res* **229**, 200-205, doi:10.1016/j.psychres.2015.07.036 (2015).

97 Okusaga, O. O. Accelerated aging in schizophrenia patients: the potential role of oxidative stress. *Aging Dis* **5**, 256-262, doi:10.14336/AD.2014.0500256 (2014).

98 Muraleedharan, A., Menon, V., Rajkumar, R. P. & Chand, P. Assessment of DNA damage and repair efficiency in drug naïve schizophrenia using comet assay. *J Psychiatr Res* **68**, 47-53, doi:10.1016/j.jpsychires.2015.05.018 (2015).

99 Anders, S. & Huber, W. Differential expression analysis for sequence count data. Genome biology 11, R106, doi:10.1186/gb-2010-11-10-r106 (2010).

100 Robinson, M. D., McCarthy, D. J. & Smyth, G. K. edgeR: a Bioconductor package for differential expression analysis of digital gene expression data. Bioinformatics 26, 139-140, doi:10.1093/bioinformatics/btp616 (2010).

101 Brumbaugh, C. D., Kim, H. J., Giovacchini, M. & Pourmand, N. NanoStriDE: normalization and differential expression analysis of NanoString nCounter data. BMC bioinformatics 12, 479, doi:10.1186/1471-2105-12-479 (2011).

102 Risso, D., Ngai, J., Speed, T. P. & Dudoit, S. Normalization of RNA-seq data using factor analysis of control genes or samples. Nature biotechnology 32, 896-902, doi:10.1038/nbt.2931 (2014).

103 Seyednasrollah, F., Laiho, A. & Elo, L. L. Comparison of software packages for detecting differential expression in RNA-seq studies. Briefings in bioinformatics 16, 59-70, doi:10.1093/bib/bbt086 (2015).

104 Soneson, C. & Delorenzi, M. A comparison of methods for differential expression analysis of RNA-seq data. BMC bioinformatics 14, 91, doi:10.1186/1471-2105-14-91 (2013).

105 Vila-Casadesus, M., Gironella, M. & Lozano, J. J. MiRComb: An R Package to Analyse miRNA-mRNA Interactions. Examples across Five Digestive Cancers. PloS one 11, e0151127, doi:10.1371/journal.pone.0151127 (2016).

Supplementary Figures

Supplementary Figure S1 | Further characterisation of drug delivery NPs. a, Adsorption efficiency of different drugs to NPs (total adsorbed drug weight to NPs/initial feeding amount of drug weight) × 100 (*n* = 3 experiments). **b,** Amount of drug (μg) loaded to 1 mg of NPs. **c,** Rate of adsorption of increasing concentrations of Gap 26 and MitoQ to 1 mg of NPs. **d,** Release with time of Gap26 and MitoQ from NPs into PBS (*n* = 3). **e,** Release with time of Gap26 from NPs into PBS or culture medium containing 10% fetal calf serum (*n* = 3 biological replicates). All measurements are means ± s.e.m.

Supplementary Figure S2 | Effects of different drug delivery-NPs on the secretions from model trophoblast barriers *in vitro*. a-d, Levels of DNA damage, as recorded by an increase in mean tail moment (product of comet length and tail intensity) using the alkaline comet assay, in fibroblasts (a,b) or dendrite lengths of dissociated cortical neurons (c,d) after exposure to conditioned media or normal tissue culture medium (Control, C). Conditioned media was collected from bilayered BeWo barriers following 24 h exposure to atmospheric oxygen (21%), hypoxia followed by partial reoxygenation (2-12%) or hypoxia followed by full reoxygenation (2-21%) (a-c), or to 30 µM benzoquinone/30 µM hydroquinone (BQ/HQ) (d). Barriers were treated with unbound NPs, drug-loaded NPs or soluble drug on its own. **e,** Dendrite lengths in dissociated cortical neurons after direct exposure to NPs with or without drug. **f-h,** Effect of direct application of unbound MitoQ, blank NPs or MitoQ-NPs on total cell number (f), neuron count (g) and astrocyte count (h) of rat cortical cultures. **i,** Cell viability of fibroblasts directly exposed to 0.5 µM or 5 µM MitoQ-NPs or NPs alone, as recorded with the Trypan blue assay. **j,** DNA damage in fibroblasts 24 h after direct exposure to BQ/HQ and unbound or drug-containing NPs. **k**, Percentage of applied FITC-BSA

detected below a bilayered BeWo grown at atmospheric oxygen (21%), hypoxia (2%) or hypoxia followed by reoxygenation (2-12%), 1 h after addition of FITC-BSA to the medium above the barrier. As a positive control, FITC-BSA was measured in the Transwell insert. Differences in concentration between the media below the barrier and the medium in the insert were significant for all oxygen conditions. Replicates (separate *in vitro* experiments) per condition: (a, b, j) $n = 9$; (c, d) $n = 15$; (e) $n = 10$ for C and C+NP, $n = 9$ for C+MitoQ; (f) $n = 43$, 5, 23, 34 from left to right; (g) $n = 44$; 5, 9, 17; (h) $n = 74$, 10, 5, 5; (i,k) $n = 3$. *$p < 0.05$, **$p < 0.01$, ***$p < 0.001$ using one-way ANOVA with Bonferroni correction for multiple comparisons. For clarity, not all significant differences are shown. All measurements are means ± s.e.m.

Supplementary Figure S3 | Further analysis of effects of drug delivery NPs *in vivo*. Effects of *in vivo* exposure to gestational normoxia (21%) or hypoxia (11%) with maternal injection of saline or MitoQ-NPs were investigated. **a,** Brain weight of offspring at gestational day 20 (top; $n = 3$ litters) or postnatal day 30 (bottom; $n = 24$ individuals). **b,** Diameter of labyrinth (top) and decidua zones (bottom) of the placenta (number of biological replicates from left to right: $n = 22$, 16, 10, 12). **c,** Confocal images of rat maternal liver and cortex after maternal injection with saline or fluorescent NPs (green), counterstained with DAPI (blue), scale bar = 100 μm. The high density of NP within the cortex, as shown in the image, was found in sparsely distributed areas throughout the maternal brain. **c,** Light micrographs showing the gross histology (H&E stain) of the placental labyrinth at GD20 following *in vivo* normoxia or hypoxia with maternal saline or MitoQ-NP injection (scale bar = 2 μm). **d,** Immunostaining of placental blood vessels in the labyrinth with anti-CD34 antibody (scale bar = 50 μm). **e,** Number of blood vessels per field of view (top; $n = 3$) and total perimeter of blood vessels, normalised to control levels, per field of view (bottom; $n =$

3). **f,** Levels of fluorescent DCF in the labyrinth (top) and the junctional zones (bottom) of the placenta (*n* = 6) as well as in cortex (top) and cerebellum (bottom) of the fetus (*n* = 6). All measurements are means ± s.e.m.

Supplementary Figure S4 | Further analysis of secretions from the placental barrier after application of drug delivery NPs. a, Total levels of small RNAs and miRNAs in media conditioned by BeWo barriers exposed to different oxygen tensions for 24 h (*n* = 3 experiments). **b,** Analysis of particle sizes and concentrations in conditioned media collected from below BeWo barriers exposed to varying levels of oxygen with or without MitoQ-NP application. The grey bar delimits particles of 50-150 nm in size, which includes the size range of exosomal vesicles. **c,** Total levels of small RNAs and miRNAs in culture media conditioned by rat placenta. Placentas had been exposed to gestational normoxia or hypoxia following maternal administration of saline or MitoQ-NPs and were subjected to 24 h exposure to 21% or 2% oxygen tension *ex vivo* (Normoxia+Saline, *n* = 3 biological replicates from different dams; all other conditions, *n* = 4). **d,** The effect of maternal MitoQ-NP injection (orange) on those miRNAs that showed significantly different levels under hypoxic conditions (blue) is depicted as log2 fold change, compared to control conditions (normoxia, no NPs). Extracted placentas were additionally exposed to 21% (top) or 2% oxygen (bottom) *ex vivo*. **e,** Levels of total amino acids in medium conditioned by rat placentas exposed to maternal normoxia, M(21%), or hypoxia, M(11%), following maternal administration of saline or MitoQ-NPs. To discover any potentially masked effects of amino acid secretion from the placenta, medium diluted 1:2 with PBS was compared with undiluted medium. All measurements are means ± s.e.m.

Supplementary Figure S5 | Analysis of fetal neurons after indirect exposure to hypoxia and drug delivery NPs. Cortical cultures analysed following exposure to media conditioned by BeWo barriers (a,c,e,g) or rat placenta (b,d,f,h). BeWo barriers had been exposed to 21%, 8% or 2-12% oxygen. Placentas had been exposed to maternal normoxia, M(21%), or hypoxia, M(11%), for 6 d with maternal saline or MitoQ-NP injection and to different oxygen levels *ex vivo* (21%, 2%). **a,b,** Length of dendritic processes (number of biological replicates from left to right: a, $n = 25, 15$; b, $n = 14, 14, 13, 20, 41, 29$). **c,d,** Process length of TH-positive cells (c, $n = 25, 15$; d, $n = 60, 30, 15, 9, 27, 15$). **e,f,** Staining intensity of GluN1 subunits, normalised to control levels (e, $n = 15, 8$; f, $n = 76, 46, 28, 36, 51, 40$). **g,h,** Ratio of astrocytes to neurons (g, $n = 25, 15$; h, $n = 20, 40, 15, 35, 47, 35$). Significant differences determined using ANOVA with Tukey's test to correct for multiple comparisons (*$p < 0.05$, **$p < 0.01$, ***$p < 0.001$). All measurements are means ± s.e.m.

Supplementary Figure S6 | Neuronal cell count after indirect exposure to maternal hypoxia and drug delivery NPs. a-e, Total cell number *in vitro* following exposure of cortical cultures to rat placenta conditioned media (a-b; number of biological replicates from left to right: a, $n = 10, 10, 8, 10$; b, $n = 23, 20, 35, 35, 41, 41$) or fetal plasma (c; $n = 10$), and *in vivo* in thalamic reticular nucleus (TRN, h; $n = 35, 51, 55, 43$) and in somatosensory, auditory and retrosplenial cortex combined (CTX, i; $n = 63, 126, 126, 98$) of the offspring. **f-j,** Number of tyrosine hydroxylase (TH)-positive neurons *in vitro* following exposure of cortical cultures to rat placenta conditioned media (f-g; f, $n = 12, 10, 8, 10$; g, $n = 5$) or fetal plasma (h; $n = 5$), and *in vivo* in TRN (i; $n = 64, 56, 60, 44$) and CTX (j; $n = 121, 108, 123, 98$) of the offspring. Offspring had been exposed *in vivo* to maternal normoxia, M(21%), or hypoxia, M(11%), along with maternal injection of saline or MitoQ-NPs. Conditioned media were obtained after *ex vivo* incubation of extracted placenta for 24 h at different oxygen

levels (8%, 21%, 2%). ANOVA with correction for multiple comparisons using Tukey's test was used to detect significant differences (*$p < 0.05$). All measurements are means ± s.e.m.

Supplementary Figure S7 | Diagram of experimental set-up and outcomes of hypoxia and MitoQ-NP injection *in vitro* and *in vivo*. Presented are the observed effects of maternal hypoxia and maternal MitoQ-NP *i.v.* injection on levels of reactive oxygen species (ROS) in fetal and maternal tissues and placenta as well as on structural and transcriptome changes in the offspring brain. Observed effects of conditioned medium from rat placenta or of fetal plasma following maternal hypoxia and MitoQ-NP exposure on cortical cultures are also shown, along with effects of conditioned medium from directly exposed BeWo barriers. Interrupted arrow indicates potential interaction between secreted miRNAs and transcriptome changes in the fetal brain.

Supplementary Tables

Supplementary Table S1 | MitoQ concentration in conditioned media. Concentration of soluble MitoQ was measured in conditioned medium collected from below BeWo barriers that had been exposed to 2-12% oxygen with or without application of 0.5 µM MitoQ-NP to the top of the barrier. MitoQ was also quantified in medium conditioned by placentas collected from dams exposed to gestational hypoxia combined with injection of MitoQ-NP (0.5 µM blood concentration) or saline. MitoQ concentration is displayed in pM and as % of original exposure. All measurements are means ± s.e.m. Significance was calculated using student's *t*-test.

Supplementary Table S2 | Amino acid concentrations in hypoxia-conditioned media from rat placenta. Placentas were exposed to maternal hypoxia combined with atmospheric oxygen conditions or 2% oxygen for 24 h *ex vivo*. Either saline or MitoQ-NP were maternally injected at the start of *in vivo* exposure. Amino acid concentrations in µmol/L (s.e.m.) were measured in the surrounding conditioned medium. $^{\#}p < 0.05$, $^{\#\#}p < 0.01$; significant compared to saline condition of the respective oxygen condition.

Supplementary Table S3 | Amino acid concentrations in hypoxia-conditioned media from rat placenta. Placenta were exposed to maternal hypoxia combined with 8% oxygen for 24 h *ex vivo*. Either saline or MitoQ-NP were maternally injected at the start of *in vivo* exposure. Amino acid concentrations in µmol/L (s.e.m.) were measured in the surrounding conditioned medium. No significant differences were detected.

Supplementary Table S4 | Amino acid concentrations in hypoxia-conditioned media from rat placenta. Placenta were exposed to maternal hypoxia combined with 21% oxygen for 24 h *ex vivo*. Either saline or MitoQ-NP were maternally injected at the start of *in vivo* exposure. Amino acid concentrations in μmol/L (s.e.m.) were measured in the surrounding conditioned medium. Culture medium was diluted with PBS prior to exposure to estimate the contribution of placenta-secreted amino acids compared to those already present in the culture medium. *$p < 0.05$; significant compared to normoxia saline condition.

Supplementary Table S5 | Enrichment of predicted mRNA targets for genes associated with schizophrenia copy number variants. Predicted targets of miRNAs that were found to be significantly up or down-regulated in rat placenta conditioned media or fetal plasma, under gestational hypoxia compared to normoxia, were analysed for enrichment of copy number variants associated with schizophrenia. Placental tissue and fetal plasma were collected following maternal exposure to hypoxia; placentas were incubated at 8%, 21% or 2% oxygen *ex vivo*. p values and p values corrected for multiple comparisons are given. $p < 0.05$ are highlighted in bold.

Supplementary Table S6 | Enrichment of predicted miRNA targets for genes associated with schizophrenia copy number variants, conditioning on a minimal gene set. Predicted targets of miRNAs that were found to be significantly up or down-regulated in rat placenta conditioned media or fetal plasma, under gestational hypoxia compared to normoxia, were analysed for enrichment of copy number variants associated with schizophrenia. Analysis was conditioned on the 'minimal' gene sets previously shown to capture CNS-related gene set enrichment. Placental tissue and fetal plasma were collected following maternal exposure

to hypoxia; placentas were incubated at 8%, 21% or 2% oxygen *ex vivo*. $p < 0.05$ are highlighted in bold.

Supplementary Table S7 | Enrichment of fetal brain transcriptome for miRNA targets. Gene transcripts that were significantly altered in the fetal brain following hypoxia were analysed for enrichment of predicted miRNA targets. Predicted targets were analysed of those miRNAs that were significantly altered in conditioned medium from rat placenta or in fetal plasma, following gestational hypoxia. *p* values were calculated using Fisher's exact test.

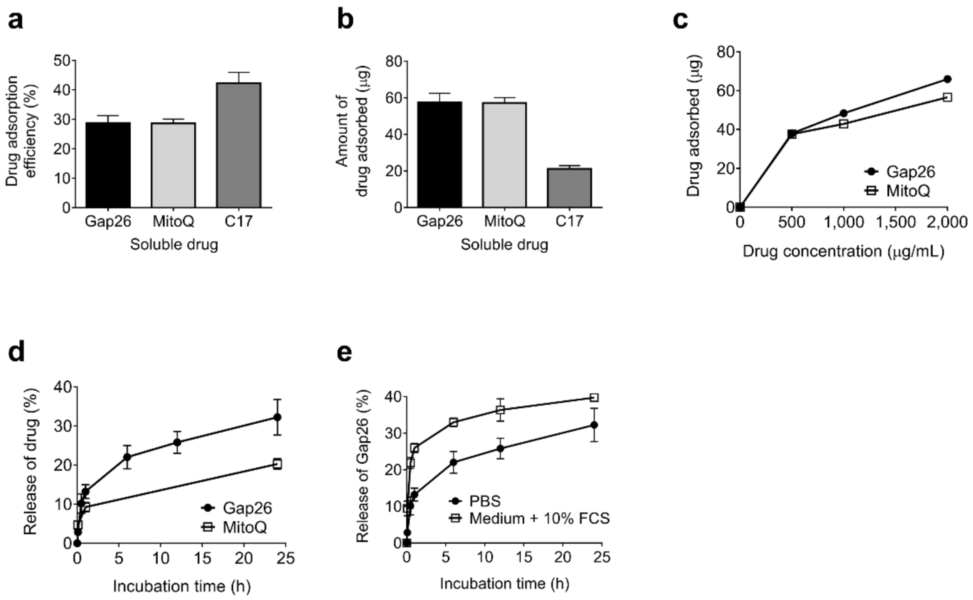

Supplementary Figure S1

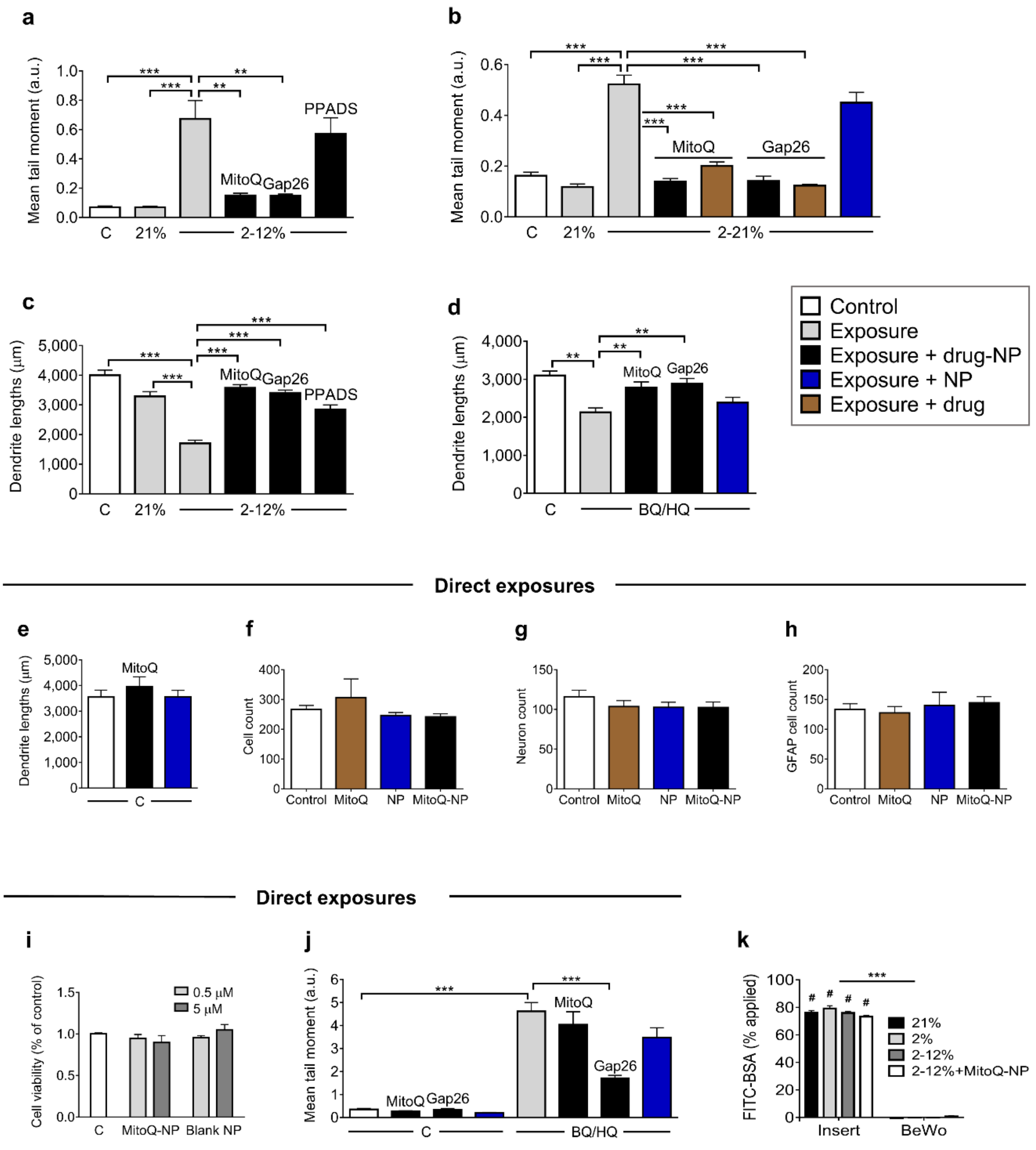

Supplementary Fig S2

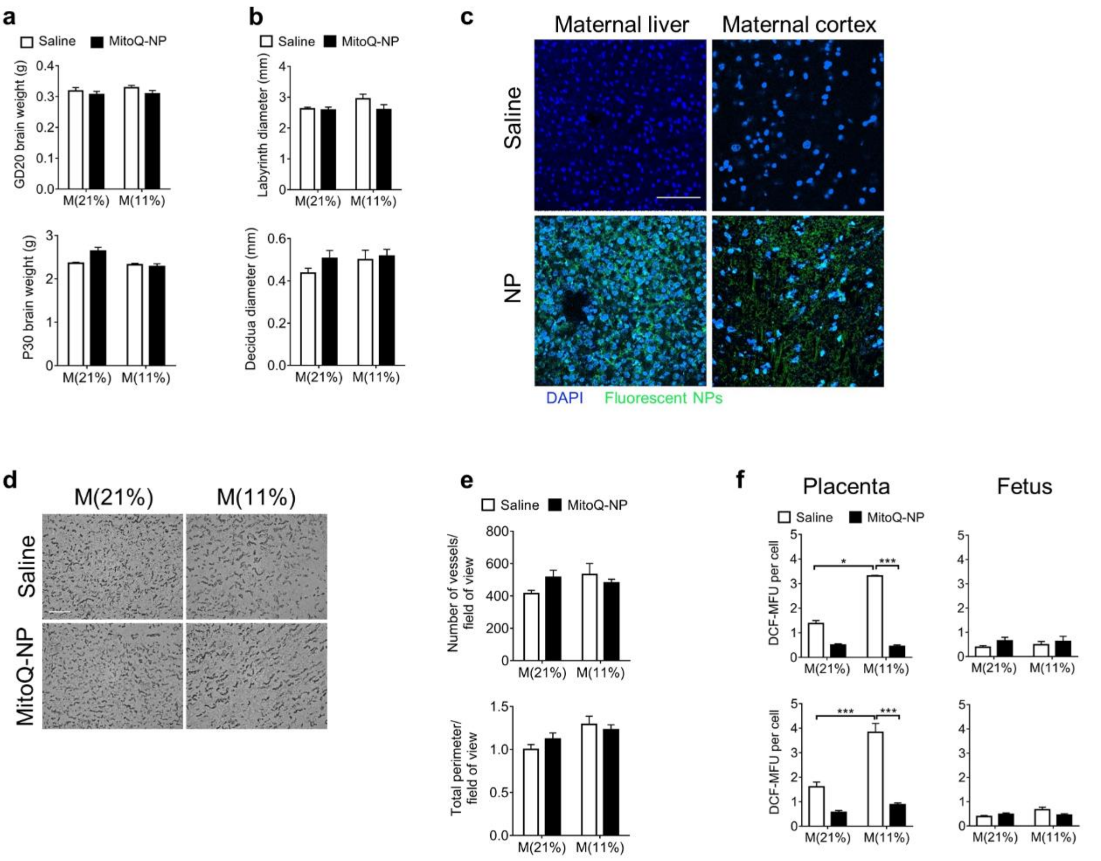

Supplementary Figure S3

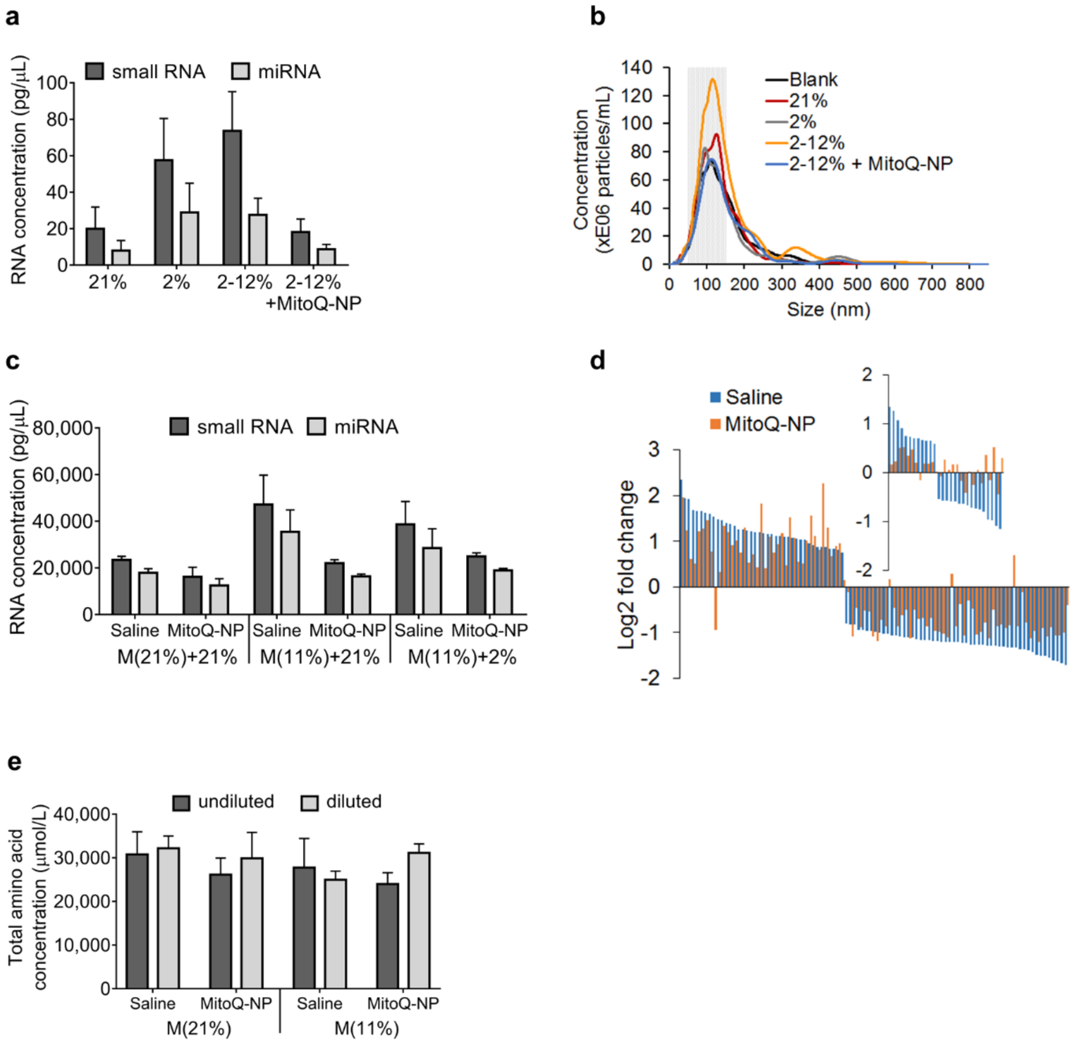

Supplementary Figure S4

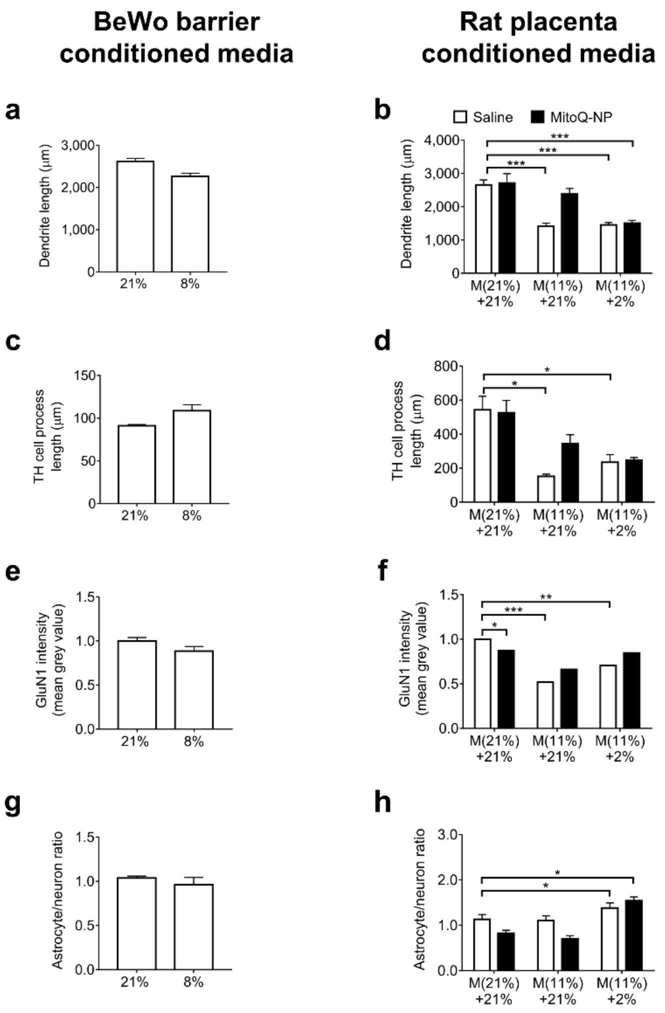

Supplementary Figure S5

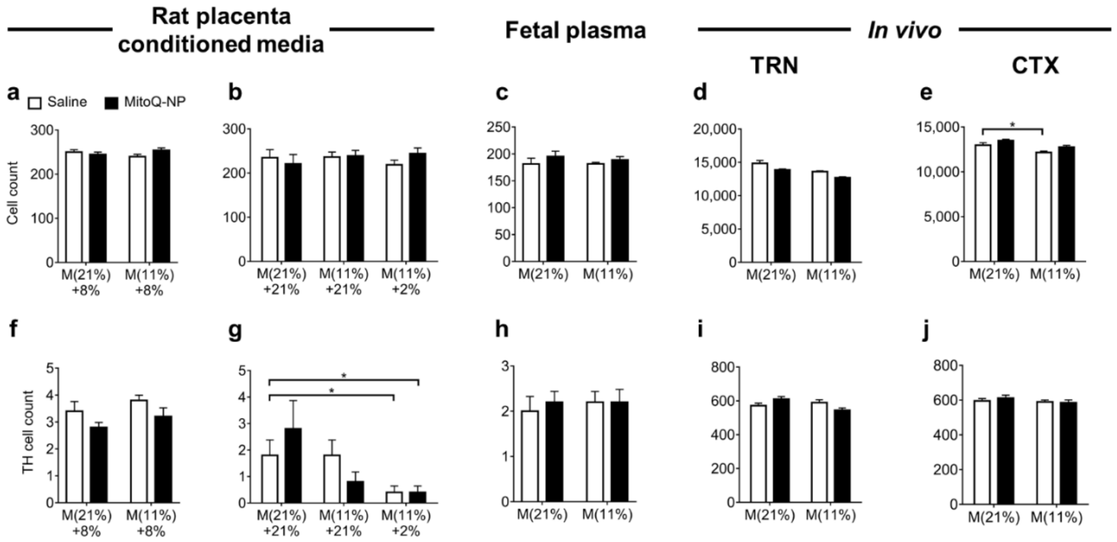

Supplementary Figure S6

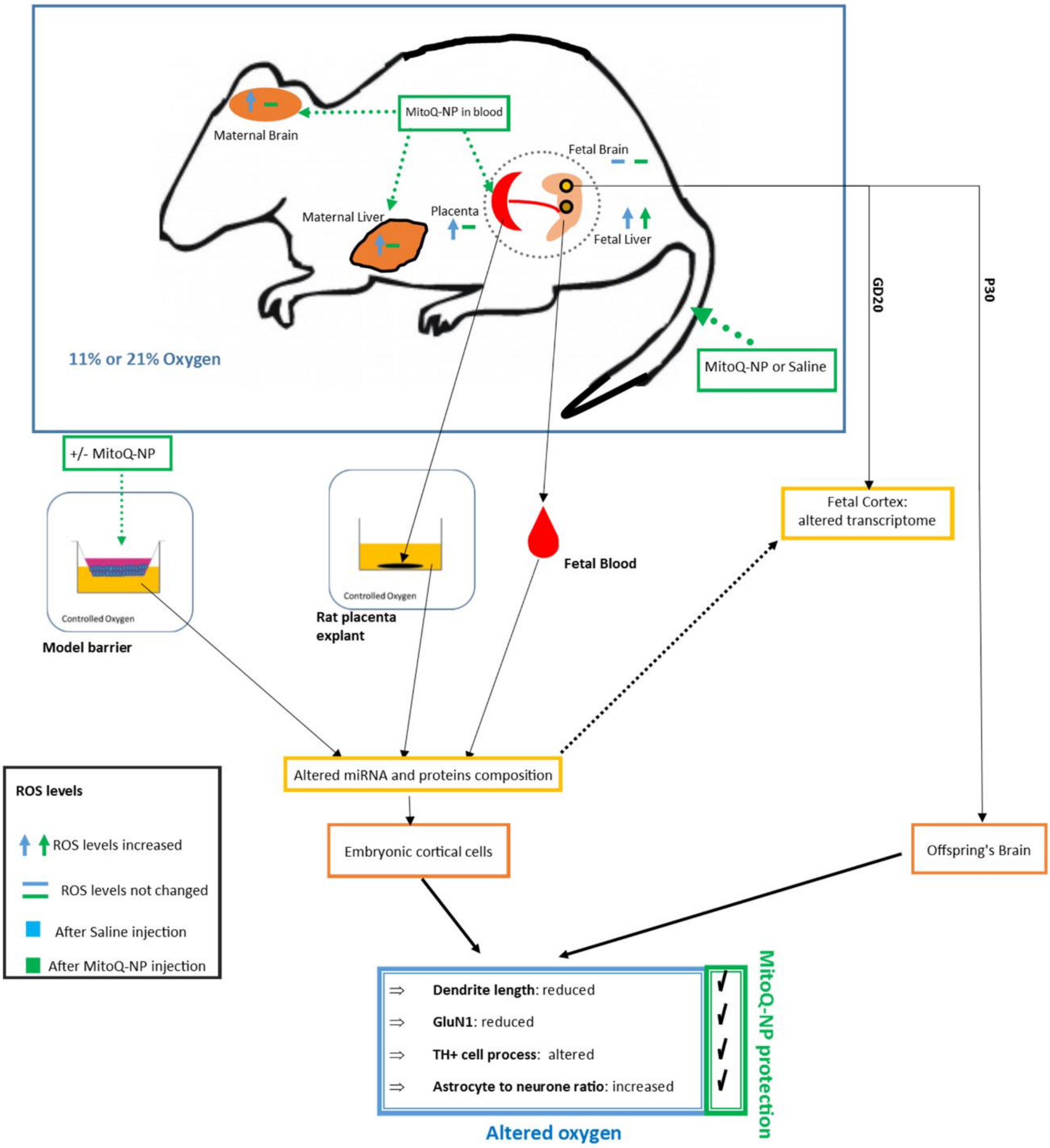

Supplementary Figure S7

Supplementary Table S1

	**Original exposure: NP-bound MitoQ in pM** (concentration above barrier or blood concentration)	**MitoQ concentration in pM (% exposure)**		
		**Saline**	**MitoQ-NP**	**Significance**
**BeWo conditioned medium**	$5x10^5$	0.32±0.21 ($6.54x10^{-7}$)	0.19±0.13 ($3.82x10^{-7}$)	ns
**Rat placenta conditioned medium**	$5x10^5$	0.11±0.01 ($2.10x10^{-7}$)	0.12±0.03 ($2.40x10^{-7}$)	ns

Supplementary Table S2

	M(21%) + 21%		M(11%) + 21%		M(11%) + 2%	
	**Saline**	**MitoQ-NP**	**Saline**	**MitoQ-NP**	**Saline**	**MitoQ-NP**
**Alanine**	1390.7 (204.6)	2118.7 (398.1)	997.7 (115.7)	1574.5# (59.5)	923.3 (75.11)	1364.0 (304.0)
**Arginine**	473.0 (132.7)	670.3 (150.4)	343.7 (94.6)	353.5 (85.6)	181.0 (63.6)	69.0 (2.8)
**Aspartate**	581.0 (26.7)	935.0# (100.8)	520.0 (91.1)	840.5 (76.5)	467.3 (29.1)	751.5 (156.5)
**Citrulline**	61.7 (4.1)	46.3 (23.5)	27.0 (22.0)	75.5 (5.5)	53.3 (22.7)	94.5 (3.5)
**Cysteine**	107.0 (31.6)	173.3 (59.6)	45.7 (4.9)	59.5 (30.5)	18.0 (6.7)	47.0 (1.0)
**Glutamate**	1712.7 (205.9)	2283.7 (421.4)	1259.3 (279.8)	2013.5 (14.5)	1285.7 (207.6)	1961.5 (336.5)
**Glutamine**	2324.0 (507.1)	2521.3 (557.1)	1319.0 (476.5)	1786.5 (36.5)	1688.3 (754.9)	1782.0 (153)
**Glycine**	1423.3 (190.5)	1916 (304.6)	1044.3 (93.2)	1492.5# (17.5)	1051.0 (58.80)	1365.5 (255.3)
**Histidine**	317.7 (25.7)	456.7 (49.7)	302.0 (6.7)	408.0# (22.0)	271.0 (1.5)	341.5 (55.5)
**Isoleucine**	942.0 (61.1)	1147.7 (105.4)	859.7 (26.1)	1036.5# (13.5)	864.0 (15.9)	1038.0 (77.0)
**Leucine**	1371.3 (112.5)	1809.0 (196.1)	1226.3 (89.5)	1609.5# (36.5)	1182.0 (19.3)	1479.0 (182.0)
**Lysine**	1332.3 (103.8)	1765.3 (128.9)	1219.3 (38.7)	353.5## (60.5)	1199.3 (3.3)	1498.0 (204.0)
**Methionine**	367.3 (41.9)	503.3 (77.5)	306.3 (20.2)	416.0# (10.0)	300.0 (7.1)	391.0 (55.0)
**Ornithine**	318.0 (91.8)	400.3 (4.7)	403.3 (84.6)	661.5 (94.5)	553.7 (28.1)	855.5 (134.5)
**Phenylalanine**	653.7 (68.0)	873.3 (105.1)	577.3 (20.2)	769.0## (8.0)	564.7 (12.5)	717.0 (95.0)
**Serine**	1201.3 (123.0)	1665.7 (279.1)	1030.7 (85.3)	1409.5# (35.5)	1007.7 (41.5)	1312.5 (217.5)
**Threonine**	1199.3 (92.1)	1558.7 (167.4)	1071.3 (47.6)	1169.0 (112.0)	1034.3 (21.2)	1208.0 (154.0)
**Tyrosine**	574.3 9 (61.0)	721.3 (89.6)	514.3 (22.2)	662.5# (3.5)	511.3 (8.3)	633.5 (72.5)
**Valine**	1178.7 (91.6)	1548.3 (163.8)	1064.7 (34.4)	1358.5 # (41.5)	1021.3 (13.3)	1270.5 (141.5)
**Total amino acids**	17529.3 (1980.0)	23114.3 (3254.6)	14132.0 (1434.1)	19295.5 (295.5)	14179.3 (1112.8)	18179.5 (2219.5)

Supplementary Table S3

	M(21%) + 8%		M(11%) + 8%	
	**Saline**	**MitoQ-NP**	**Saline**	**MitoQ-NP**
**Alanine**	3155.5 (729.9)	2428.9 (461.6)	2757.6 (758.8)	2391.5 (272.2)
**Arginine**	973.4 (250.8)	560.0 (79.2)	574.0 (307.0)	691.3 (108.3)
**Asparagine**	727.2 (216.9)	512.0 (92.1)	648.5 (265.5)	490.4 (40.5)
**Aspartate**	990.9 (245.8)	760.9 (171.8)	919.0 (368.1)	870.1 (60.1)
**Citrulline**	117.5 (8.0)	145.2 (28.4)	148.5 (51.5)	103.9 (16.8)
**Cysteine**	294.9 (63.6)	223.2 (25.7)	257.0 (48.8)	223.6 (16.1)
**Glutamate**	2842.4 (499.1)	2551.2 (449.3)	2496.4 (558.7)	2160.7 (214.0)
**Glutamine**	2965.5 (335.4)	2413.9 (111.1)	2502.4 (179.1)	2007.1 (280.7)
**Glycine**	2407.2 (365.8)	2109.7 (299.2)	2226.5 (472.6)	2035.6 (278.8)
**Histidine**	541.0 (133.0)	421.0 (81.8)	387.8 (60.9)	319.2 (33.3)
**Isoleucine**	780.5 (363.3)	1199.5 (128.3)	1281.7 (233.7)	1014.6 (94.9)
**Leucine**	1908.6 (51.0)	2019.2 (304.0)	2220.7 (220.6)	1905.8 (186.9)
**Lysine**	2253.1 (437.1)	1879.7 (269.3)	2041.1 (437.0)	1648.5 (178.6)
**Methionine**	618.9 (124.9)	518.4 (80.4)	539.8 (128.8)	443.8 (37.3)
**Ornithine**	188.9 (31.2)	244.6 (59.7)	325.2 (120.0)	209.6 (44.6)
**Phenylalanine**	637.7 (303.7)	975.9 (137.1)	1054.2 (235.9)	893.8 (84.7)
**Proline**	1511.0 (316.6)	1174.7 (255.6)	1045.1 (670.3)	1208.6 (162.0)
**Serine**	2138.1 (461.9)	1739.7 (282.9)	1938.4 (547.7)	1677.1 (235.9)
**Threonine**	1912.6 (321.8)	1592.5 (191.5)	1698.6 (326.6)	1453.5 (143.4)
**Tryptophan**	120.5 (83.4)	158.8 (83.0)	46.3 (46.3)	0 (0)
**Tyrosine**	1675.8 (933.1)	783.6 (104.0)	827.7 (152.3)	683.3 (62.8)
**Valine**	2012.0 (388.6)	1695.5 (226.3)	1823.9 (386.8)	1553.2 (167.3)
**Total amino acids**	30773.27 (5199.8)	26137 (3811.1)	27760.7 (6681.3)	23985.3 (2612.3)

Supplementary Table S4

	M(21%) + 21%		M(11%) + 21%	
	**Saline**	**MitoQ-NP**	**Saline**	**MitoQ-NP**
**Alanine**	4284.9 (539.6)	3042.4 (720.6)	2661.6* (137.9)	3367.3 (219.1)
**Arginine**	423.7 (193.3)	802.2 (158.7)	503.7 (250.0)	829.1 (180.3)
**Asparagine**	564.4 (316.0)	635.7 (199.2)	526.4 (123.6)	477.4 (244.1)
**Aspartate**	406.6 (271.5)	944.5 (263.1)	749.5 (94.8)	809.7 (204.3)
**Citrulline**	20.0 (12.8)	38.0 (38.0)	157.7 (28.4)	132.6 (24.4)
**Cysteine**	174.3 (57.8)	241.3 (26.2)	307.6 (28.4)	271.6 (68.0)
**Glutamate**	3182.4 (285.0)	2513.1 (470.2)	2241.5 (193.1)	2672.6 (217.1)
**Glutamine**	2898.1 (258.7)	3058.0 (407.3)	2044.8 (259.4)	3179.8 (548.9)
**Glycine**	2818.4 (262.1)	2436.0 (453.3)	2250.2 (101.3)	2611.6 (186.7)
**Histidine**	713.3 (133.0)	541.0 (144.4)	479.7 (47.9)	613.3 (45.3)
**Isoleucine**	1338.7 (226.5)	1300.7 (240.8)	956.0 (73.8)	1305.2 (73.1)
**Leucine**	2876.5 (502.3)	2267.9 (480.3)	1955.9 (158.4)	2438.1 (160.1)
**Lysine**	2543.9 (445.0)	2043.7 (421.5)	1838.8 (147.2)	2195.6 (150.7)
**Methionine**	720.5 (142.4)	582.8 (127.8)	446.3 (37.2)	573.1 (29.7)
**Ornithine**	230.8 (49.3)	322.8 (96.5)	406.9 (180.7)	301.7 (66.0)
**Phenylalanine**	1374.4 (246.9)	1085.2 (221.0)	929.4 (74.4)	1116.2 (68.8)
**Proline**	1876.6 (305.5)	1518.1 (352.4)	1313.2 (63.5)	1573.0 (123.9)
**Serine**	893.4 (579.8)	1966.5 (433.6)	1237.5 (593.7)	1730.5 (441.6)
**Threonine**	1540.4 (132.1)	1806.8 (282.1)	1621.3 (139.2)	1835.5 (118.0)
**Tryptophan**	202.0 (117.2)	46.6 (46.6)	161.1 (24.8)	230.4 (37.5)
**Tyrosine**	913.3 (171.1)	837.3 (152.4)	603.0 (234.4)	884.5 (65.3)
**Valine**	2186.3 (343.4)	1856 (352.9)	1570.0 (115.3)	2005.5 (119.0)
**Total amino acids**	32182.9 (2845.4)	29886.4 (5953.3)	24962.0 (1994.3)	31154.2 (2047.3)

Supplementary Table S5

		Combined		Deletion		Duplication	
	**# Genes**	***p***	***p* corr**	***p***	***p* corr**	***p***	***p* corr**
Rat placenta CM (8%)							
**All**	3083	**3.39E-05**	**1.22E-03**	1.07E-01	1.00E+00	**1.26E-04**	**4.55E-03**
**Down**	1565	**1.77E-03**	6.36E-02	**9.58E-03**	3.45E-01	**3.38E-02**	1.00E+00
**Up**	2005	**1.08E-04**	**3.87E-03**	1.14E-01	1.00E+00	**5.00E-04**	**1.80E-02**
Rat placenta CM (21%)							
**All**	1495	**3.95E-03**	1.42E-01	**1.69E-02**	6.07E-01	6.00E-02	1.00E+00
**Down**	665	5.43E-01	1.00E+00	3.63E-01	1.00E+00	7.93E-01	1.00E+00
**Up**	917	**6.72E-04**	**2.42E-02**	**1.78E-03**	6.41E-02	**3.09E-02**	1.00E+00
Rat placenta CM (2%)							
**All**	2895	**3.02E-04**	**1.09E-02**	**7.55E-05**	**2.72E-03**	9.07E-02	1.00E+00
**Down**	771	**1.06E-02**	3.82E-01	**2.30E-05**	**8.30E-04**	6.02E-01	1.00E+00
**Up**	2400	**3.05E-05**	**1.10E-03**	**3.70E-05**	**1.33E-03**	**2.72E-02**	9.80E-01
Fetal plasma							
**All**	1265	**1.07E-02**	3.84E-01	**1.18E-03**	**4.23E-02**	3.54E-01	1.00E+00
**Down**	599	5.15E-02	1.00E+00	**2.43E-04**	**8.74E-03**	7.00E-01	1.00E+00
**Up**	727	**3.50E-02**	1.00E+00	1.19E-01	1.00E+00	1.40E-01	1.00E+00

Supplementary Table S6

		Combined		Deletion		Duplication	
	**# Genes**	**coeff**	***p* value**	**coeff**	***p* value**	**coeff**	***p* value**
Rat placenta CM (8%)							
**All**	3083	+	**5.18E-03**	+	1.27E-01	+	**1.21E-02**
**Down**	1565	+	**1.99E-02**	+	**4.23E-02**	+	1.25E-01
**Up**	2005	+	**2.01E-03**	+	7.43E-02	+	**1.00E-02**
Rat placenta CM (21%)							
**All**	1495	+	**3.62E-02**	+	6.12E-02	+	1.81E-01
**Down**	665	-	5.75E-01	-	8.32E-01	-	6.28E-01
**Up**	917	+	**1.32E-02**	+	**7.45E-03**	+	1.96E-01
Rat placenta CM (2%)							
**All**	2895	+	**1.47E-02**	+	**3.38E-02**	+	2.28E-01
**Down**	771	+	1.01E-01	+	1.43E-01	-	5.93E-01
**Up**	2400	+	**1.64E-03**	+	**9.63E-03**	+	6.96E-02
Fetal plasma							
**All**	1265	+	8.59E-02	+	5.29E-02	-	5.24E-01
**Down**	599	+	5.41E-02	+	**1.14E-02**	-	5.31E-01
**Up**	727	+	2.85E-01	+	3.30E-01	+	4.48E-01

Supplementary Table S7

	% targets of significant genes	% targets of all genes	Fold enrichment	*p* value
**Rat placenta conditioned medium**	12.72	11.04	1.15	0.003
**Fetal plasma**	10.53	9.28	1.13	0.013
